# Material-Driven Therapeutics: Functional Nanomaterial Design Paradigms Revolutionizing Osteosarcoma Treatment

**DOI:** 10.3390/jfb16060213

**Published:** 2025-06-05

**Authors:** Zewei Zhang, Fang He, Wenqu Li, Beibei Liu, Cheng Deng, Xiaojuan Qin

**Affiliations:** 1 Department of Ultrasound Medicine, Union Hospital, Tongji Medical College, Huazhong University of Science and Technology, Wuhan 430022, China; m202376284@hust.edu.cn (Z.Z.); m202276082@hust.edu.cn (F.H.); lwq0918@hust.edu.cn (W.L.); bellaliu@hust.edu.cn (B.L.); 2 Clinical Research Center for Medical Imaging in Hubei Province, Wuhan 430022, China; 3 Hubei Province Key Laboratory of Molecular Imaging, Wuhan 430022, China; 4 Department of Medical Ultrasound, Tongji Hospital, Tongji Medical College, Huazhong University of Science and Technology, Wuhan 430030, China; 5 Hubei Province Key Laboratory of Precision Radiation Oncology, Wuhan 430022, China

**Keywords:** osteosarcoma, nanomaterials, treatment, malignant bone tumor

## Abstract

Osteosarcoma (OS), a prevalent primary malignant bone tumor in children and adolescents, has maintained consistent treatment protocols since the 1970s combining surgery, chemotherapy, and radiotherapy. While effective for localized tumors, these strategies show limited efficacy against metastatic or recurrent cases. Although emerging immunotherapies (PD-1 inhibitors, CAR-T-cell therapy) demonstrate therapeutic potential, their clinical impact remains constrained by the tumor’s low immunogenicity and immunosuppressive microenvironment, resulting in suboptimal response rates. The disease’s aggressive nature and propensity for pulmonary metastasis contribute to poor prognosis, with survival rates showing negligible improvement over five decades despite therapeutic advances, creating substantial clinical and socioeconomic challenges. Recent developments in nanomedicine offer promising solutions for OS treatment optimization. This review systematically examines nanomaterial applications in OS therapy through a materials science lens, analyzing mechanism-specific interventions and highlighting notable advancements from the past five years. We critically evaluate current strategies for enhancing therapeutic efficacy while reducing toxicity profiles, ultimately outlining translational pathways and key challenges in clinical adaptation. The analysis establishes a framework for developing next-generation nanotherapeutic platforms to address persistent limitations in OS management.

## 1. Introduction

Osteosarcoma (OS) is a primary malignant bone tumor arising from malignantly transformed mesenchymal stromal cells. The annual incidence of OS in the general population ranges from two to three cases per million individuals, with a distinct peak observed during adolescence [1]. Notably, the 15–19 age group demonstrates the highest disease burden, exhibiting an annual incidence rate of 8–11 cases per million population and it is the third most common cancer affecting children and adolescents, following lymphoma and brain tumors [2]. OS arises from genetic predispositions (TP53/RB1/BLM mutations), Paget’s disease-related bone remodeling, and radiation-induced DNA damage, with SV40 viral co-detection requiring further validation [3]. Pathologically, it manifests as metaphyseal lesions with aggressive invasion, malignant osteoid production, and characteristic molecular aberrations (chromosomes 6/8 anomalies). Treatment combines margin-negative surgery with platinum-based chemotherapy, supplemented by emerging immunotherapies (checkpoint inhibitors/CAR-T-cell therapy) and targeted agents against angiogenesis and mammalian target of rapamycin (mTOR) pathways [4]. Since the introduction of chemotherapy for OS treatment in the 1970s, the 5-year survival rate of OS patients has significantly improved, reaching 60–70% [5]. Currently, the standard clinical treatment approach involves preoperative neoadjuvant chemotherapy, surgical resection, and postoperative adjuvant radiotherapy [6]. However, these treatments are associated with numerous issues. For example, there is the problem of chemotherapy resistance, as well as systemic toxicities. Additionally, postoperative recurrence is a concern, along with poor prognosis and surgically induced bone defects. These problems severely impact patients’ quality of life. To make matters worse, approximately 15–20% of OS patients have metastases at the time of diagnosis, with the lungs being the most common site (85%) and bones accounting for 8–10% [2]. The presence of metastases is a clear indicator of a poor prognosis. Specifically, the 5-year survival rate for patients with worsening lung metastases is less than 20% [7]. Therefore, there is an urgent need for a treatment that is more effective and less toxic in order to improve OS outcomes and extend patients’ life expectancy.

**Figure 1:**
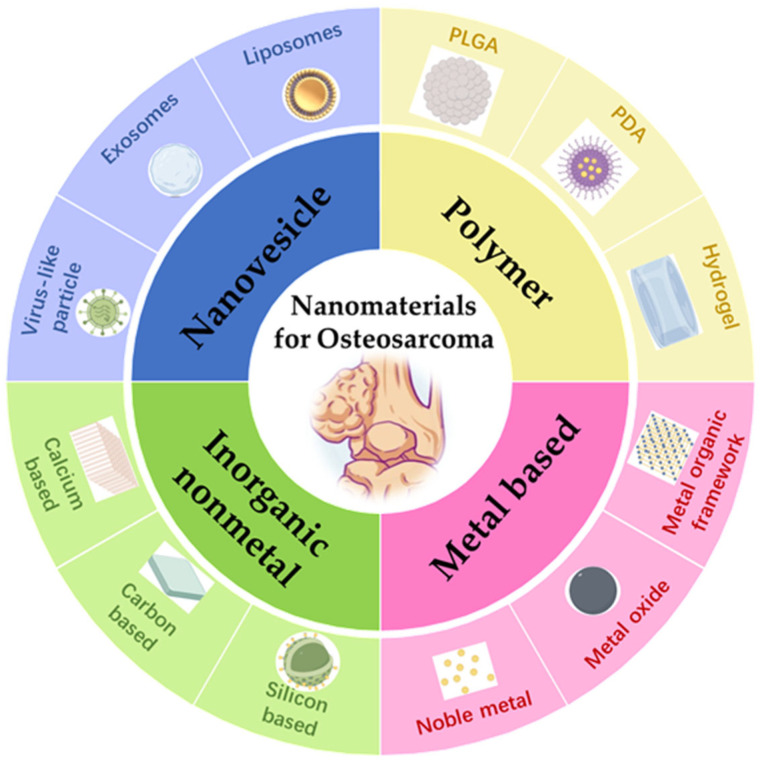
Nanomaterials for the treatment of OS.

With advancements in nanotechnology within the medical field, the emergence of nanomaterials (Figure 1) has introduced a new direction in the treatment of OS. Leveraging their unique small size, charge characteristics, and targeting capabilities, nanomaterials have found extensive applications in the treatment of various tumors, including both solid and hematologic malignancies [8]. Nanomaterials demonstrate several notable advantages in tumor therapy: (1) Nanomaterials leverage the enhanced permeability and retention (EPR) effect for passive tumor targeting, enabled by structural abnormalities in tumor vasculature. Tumor blood vessels exhibit enlarged endothelial gaps (100–1000 nm) compared to healthy tissues, permitting selective accumulation of optimally sized nanomaterials (10–200 nm) through vascular leakage. Prolonged retention arises from deficient lymphatic drainage in tumors. Key determinants include vascular permeability, interstitial pressure gradients, and nanomaterial characteristics: size extremes impair vascular extravasation or accelerate systemic clearance, while surface PEGylation enhances circulation time by evading reticuloendothelial uptake. Clinically exemplified by doxorubicin-loaded liposomes (Doxil), this strategy concentrates chemotherapeutics in malignancies like ovarian and breast cancers, optimizing therapeutic efficacy while minimizing systemic toxicity [9]. (2) Active targeting of nanomaterials integrates molecular recognition, environmental responsiveness, bioinspired engineering, and physical guidance to optimize therapeutic precision. Ligand-functionalized systems exploit tumor-specific biomarkers through antibody conjugation (e.g., HER2-targeting trastuzumab) or receptor-binding motifs (folic acid/RGD peptides) for selective accumulation. Smart nanomaterials achieve spatiotemporal control via pH-triggered drug release in acidic microenvironments or enzyme-activated payload deployment through MMP-responsive mechanisms. Biomimetic strategies employ tumor cell membrane coatings for homologous targeting or engineer immune cell-derived surfaces (e.g., T-cell membranes with PD-1 blockers) to modulate antitumor immunity. Magnetic guidance systems synergize with external fields to enhance deep-tissue penetration, exemplified by 10-fold drug concentration increases achieved in leukemia models through magnetically directed nanocarriers. These multimodal approaches collectively enhance tumor-specific delivery while circumventing systemic toxicity [10]. (3) Stimuli-responsive nanomaterials enable precise tumor therapy through microenvironmental or external activation. pH-sensitive carriers release drugs via acidic tumor-triggered structural changes, while redox-responsive systems exploit intracellular glutathione to cleave disulfide/selenium bonds. Enzyme-activated platforms utilize tumor-overexpressed proteases to degrade peptide linkers, exposing targeting ligands. Light-responsive designs combine photodynamic/photothermal effects with controlled drug release through light-induced reactive oxygen species (ROS) generation or nanostructural disruption. Temperature-sensitive materials achieve spatiotemporal control via thermal phase transitions in hydrogels or liposomes, synchronizing drug release with localized hyperthermia. These intelligent systems collectively enhance therapeutic specificity by coupling drug activation with pathological or physical triggers [11]. (4) Nanomaterials possess a large specific surface area, enabling them to carry a higher load of drug molecules and improve drug delivery. Additionally, they provide protection for encapsulated drugs, genes, and therapeutic agents, shielding them from degradation in complex physiological microenvironments and ensuring their stability and effectiveness [8]. (5) By adjusting their internal properties and applying surface modifications, nanomaterials can be loaded with functional molecules such as contrast agents, chemotherapeutic drugs, phototherapeutic agents, Fenton agents, fluorescent markers, and immunomodulators [10]. This enables the integration of diverse functions, including photothermal therapy, fluorescence imaging, and immunotherapy, facilitating multimodal synergistic treatments or combining diagnostics and therapy on a single platform. Nanomaterial-based targeting systems enhance OS treatment through controlled drug distribution and release yet face multidimensional challenges. Functionalized carriers like alendronate-conjugated liposomes significantly improve tumor accumulation (demonstrated by Morton’s orthogonal targeting system), reducing systemic exposure while enhancing local efficacy. Sustained-release platforms (e.g., silk fibroin composites) maintain therapeutic concentrations, and multifunctional theranostic systems integrate targeting, drug delivery, and imaging capabilities. However, clinical translation is hindered by manufacturing complexities, material instability, and biosafety concerns, including immunogenicity (silk fibroin) and cationic polymer cytotoxicity. Targeting efficiency is further compromised by off-organ sequestration and tumor microenvironment heterogeneity. While these systems drive advancements in personalized therapeutic strategies, critical gaps persist in understanding long-term biodistribution risks and optimizing biocompatibility for clinical implementation [12].

Despite advances in OS nanotherapeutics and multimodal strategies, clinical translation remains hindered by inadequate integration of nanomaterial innovations with OS pathobiology. While nanomaterials leverage enhanced permeability, active targeting, and stimulus responsiveness, their systematic alignment with OS-specific challenges—metastatic dissemination, immunosuppressive bone niches, and adaptive chemoresistance—lacks critical analysis. Current reviews disproportionately emphasize isolated nanomaterial functions (drug delivery, imaging) over multimodal synergy and neglect translational barriers: tumor heterogeneity-compromised targeting, undefined long-term biosafety, and scalability constraints. This gap necessitates frameworks bridging nanotherapeutic design with OS biological complexity and industrial-scale feasibility. This review establishes three translational objectives: (1) Mechanistic alignment of nanomaterial properties with OS pathobiology—hypoxic niches, aberrant angiogenesis, and metastatic pathways—to derive precision design principles; (2) systematic evaluation of pharmacological/immunological manufacturing barriers through safety-optimized production frameworks; (3) biocompatibility-driven integration of AI-optimized carriers, biomimetic interfaces, and in situ biosensing technologies, coupled with ethical precision medicine paradigms to accelerate clinical translation. The tripartite strategy bridges molecular insights with industrial-scale therapeutic development for localized and metastatic OS. This review aims to bridge the molecular mechanism/clinical reality gap, drive next-generation nanotherapeutic development, overcome traditional efficacy/toxicity trade-offs, and ultimately improve survival outcomes for localized and metastatic OS patients.

## 2. Functional Properties of Nanomaterials

### 2.1. Drug Delivery

#### 2.1.1. Controlled-Release

**Figure 2:**
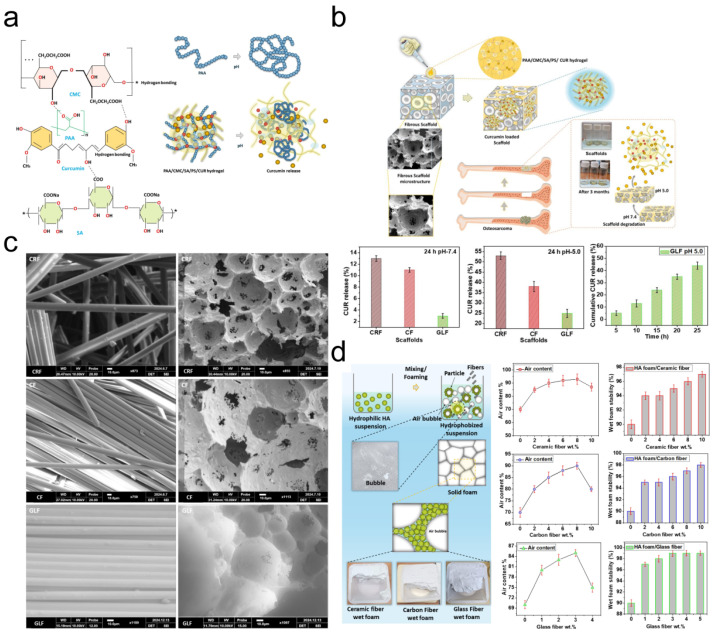
(**a**) Proposed schematic representation of curcumin delivery from PAA/SA/CMC/PS/CUR hydrogel. * means hydrogen bonding. (**b**) Curcumin release from the double-network hydrogel loading CRF, CF, and GLF scaffolds. Experiments were performed in triplicate at pH 5.0 and 7.4. (**c**) SEM images of fiber-reinforced scaffolds. (**d**) Wet foam characterization. Adapted with permission from Ref. [13]. Copyright 2025 Elsevier.

**Figure 3:**
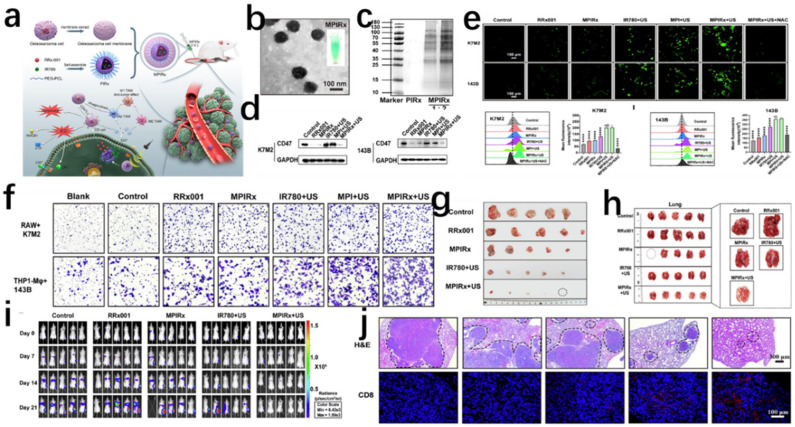
(**a**) Construction of MPIRx nanodrugs for CD47 immune checkpoint/sonodynamic therapy of osteosarcoma and pulmonary metastasis. (**b**) TEM image of MPIRx nanodrugs. (**c**) Coomassie Brilliant Blue staining of MPIRx nanodrugs. MPIRx performed in the assay at mass ratio of 1:2. (**d**) Western blot detected the CD47 protein expression in OS cells. (**e**) Fluorescence microscopy images and flow cytometry analysis of ROS in K7M2 and 143B cells which were stained with DCFH-DA after different treatments, including control, RRx001 or MPIRx only, IR780,MPI with US or MPIRx with US activation. **** indicates significant difference, NS means no significant difference. (**f**) Transwell migration assay of macrophages (THP1-Mφ, RAW264.7) cocultured with OS cells (K7M2, 143B). (**g**) Photographs of tumor tissues from different treatments including control, RRx001 or MPIRx only, IR780 or MPIRx with US activation. (**h**) Photographs of lungs from each group showing the tumor metastasis. (**i**) Bioluminescence of mouse tumor cells showing the tumor developments at orthotopic right tibia and potential lungs at 0–21 days after the first treatment. (**j**) Representative H&E staining images of pulmonary metastasis (marked with black dotted line) from each group. Adapted with permission from Ref [15]. Copyright 2023 Elsevier.

**Figure 4:**
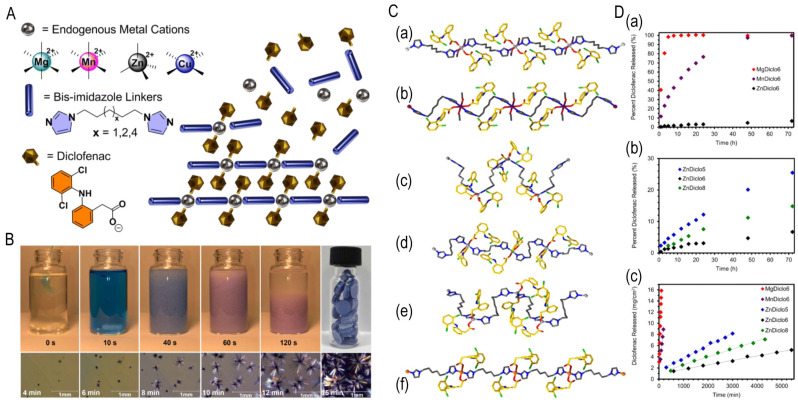
(**A**) Components of TCPs (MDicloX): M = Mg^2+^, Mn^2+^, Zn^2+^, or Cu^2+^ metal cations. Diclo = diclofenac anions, and X = bisimidazole linkers with total carbon chain lengths of 5, 6, or 8 between imidazoles. Representation of drug release from TCPs via degradation of metal–ligand interactions. (**B**) Rapid synthesis of TCP CuDiclo6 upon addition of Cu(NO_3_)_2_ in MeOH with pressed monoliths depicted and formation of large single crystals at lower concentration in MeOH. (**C**) Single-crystal structures of TCPs: (a) MgDiclo6, (b) MnDiclo6, (c) ZnDiclo5, (d) ZnDiclo6, (e) ZnDiclo8, and (f) CuDiclo6. Mg = light grey, Mn = purple, Zn = grey, Cu = orange, N = blue, O = red, Cl = green; bisimidazole and diclofenac C-atoms are shown in silver and gold, respectively. (**D**) Drug release profiles from 5 mm TCP monoliths in 0.05 M phosphate buffer, pH 6.8 at 37 °C. (a) MgDiclo6 (red), MnDiclo6 (purple), and ZnDiclo6 (black) demonstrate effect of varying metal ion composition, (b) ZnDiclo5 (blue), ZnDiclo6 (black), and ZnDiclo8 (green) demonstrate effect of varying linker composition, (c) intrinsic dissolution release (IDR) rates of diclofenac from TCPs measured using a Woods apparatus, demonstrating prolonged zero-order release kinetics. Adapted from Ref. [19].

The controlled release properties of nanomaterials enable precise spatiotemporal regulation of therapeutic agent delivery through material design, demonstrating significant potential in OS treatment through four primary mechanisms:

(1) Temporal release control: Nanocarriers modulate release kinetics through material degradation rates, drug encapsulation architectures, or chemical bond responsiveness. pH-sensitive hydrogels achieve gradual chemotherapeutic release in acidic tumor microenvironments. Fatema et al. pH-responsive hydrogel employs dual mechanisms for tumor-targeted curcumin release: PAA-based networks protonate in acidic tumor microenvironments (pH 5.0), contracting to expel drugs while deprotonating at physiological pH (7.4) to sustain release (Figure 2). The SA-reinforced dual-network architecture ensures mechanical stability. Acidic conditions significantly enhanced cumulative drug release (44% vs. 5% at 24 h), with GLF scaffolds showing 25% vs. 3% release between pH 5.0 and 7.4. This pH-dependent system enables sustained tumor-specific drug delivery for postoperative residual cancer suppression [13], while glutathione (GSH)-responsive nanomotors trigger payload release upon encountering elevated intracellular GSH levels [14].

(2) Stimulus-responsive release: As illustrated in Figure 3, endogenous-responsive nanomaterials are designed to react to intrinsic physiological conditions arising from normal biological processes or pathological alterations. These materials respond to inherent biological signals without requiring external stimulation, with representative categories including pH-responsive, redox-responsive, enzyme-responsive, and temperature-responsive systems. In contrast, exogenous-stimulus-responsive nanomaterials exhibit controlled behavioral modifications under artificially imposed physical, chemical, or biological interventions. These externally applied stimuli (typically operator-regulated) enable precise modulation of material properties and functions, with principal activation modalities encompassing optical irradiation, magnetic field application, ultrasonic excitation, and electric field manipulation [15]. Polydopamine nanoparticles release DOX under NIR irradiation, and Huang et al. bone-targeting system integrates triple stimuli-responsiveness: acidic pH-triggered DOX release via PDA protonation; reductive microenvironment-activated disulfide cleavage; and NIR-induced photothermal release. Experimental results demonstrated enhanced pharmacokinetics with prolonged circulation and amplified therapeutic efficacy through synergistic multi-stimuli drug liberation [16], while ROS-responsive carriers activated payload discharge in tumor-specific oxidative microenvironments [17].

(3) Zero-order sustained release: Structural engineering of polymer microgels [18] and nonporous coordination polymers [19] achieves constant release rates, eliminating initial burst effects and prolonging therapeutic duration. As shown in Figure 4, Murphy et al. developed therapeutic coordination polymers and nonporous materials enabling drug release via metal–ligand bond degradation rather than diffusion. With >62% drug loading and zero-order kinetics spanning three orders of magnitude (0.815–958 mg·cm^−2^·min^−1^), prolonged release is sustained. Release rates are tunable through (1) metal center selection (Mg^2^⁺, Mn^2^⁺, Zn^2+^; correlating with Lewis acidity), (2) bisimidazole ligand chain-length modifications altering crystal packing, and (3) competitive chelators (e.g., Na_2_EDTA) triggering accelerated stimulus-responsive release [19].

(4) Sequential multidrug release: Composite nanosystems like FSD-CHR@PPP [20] enable timed release of chemotherapeutics and genetic agents, amplifying synergistic effects between DNA damage and immune activation [21].

#### 2.1.2. Target-Oriented

**Figure 5:**
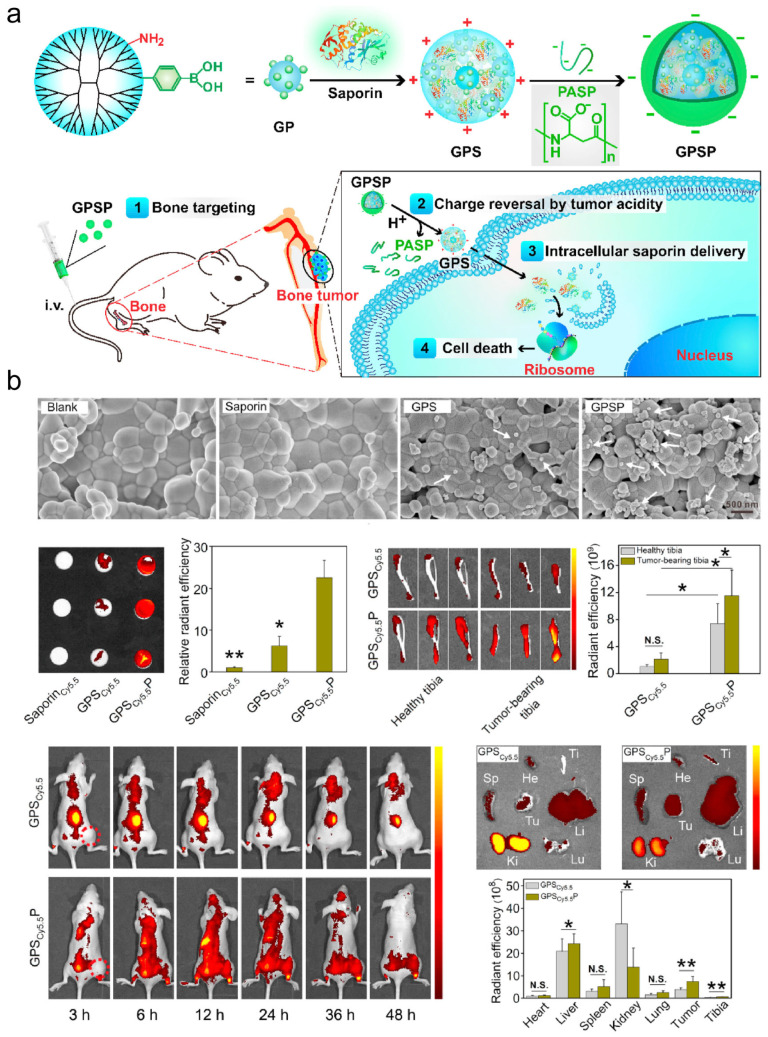
(**a**) Design of bone-targeted protein nanomedicine for the treatment of malignant bone tumors. (**b**) Bone-targeting capability of GPSP. The white arrows indicate GPS or GPSP nanoparticles adsorbed on the hydroxyapatite tablets. (* *p* < 0.05 and ** *p* < 0.01 analyzed by student’s *t*-test, N.S. means no significance, one tailed). Adapted from Ref. [23].

The targeting capability of nanomaterials refers to their ability to specifically deliver therapeutic agents to diseased tissues or cells, enhancing therapeutic efficacy while minimizing off-target effects. This targeting precision is achieved through three principal strategies:

(1) Active Targeting: Surface modification with targeting ligands (antibodies, peptides, or small molecules) enables specific recognition and accumulation at OS-specific surface markers or within tumor microenvironments. (1) Bone targeting: Phytic acid (PA)-functionalized nanoparticles (e.g., MnO_2_-PA) exploit high hydroxyapatite affinity for osseous tumor accumulation [22]. Bone-specific targeting is exemplified by saporin-boronated nanotherapeutics, which achieve ribosomal inactivation through hydroxyapatite binding, offering a targeted approach for bone malignancies [23] (Figure 5). (2) Tumor vascular targeting: iRGD peptide conjugation facilitates αvβ3 integrin-mediated tumor vascular endothelial binding and enhanced vascular penetration [24,25]. (3) Metabolic targeting: Nanoparticles targeting critical signaling molecules (STAT3, METTL3) disrupt tumor proliferation and chemoresistance pathways [26,27]. (2)Passive Targeting: Exploitation of the EPR effect enables selective nanoparticle accumulation in OS through leaky tumor vasculature [28]. (3) Bioinspired Targeting: Biomimetic nanocarriers (cell membrane-coated or bacterial outer membrane vesicles) evade immune clearance while enhancing tumor homing [17,29].

### 2.2. Immune Regulation

#### 2.2.1. Targeted Modulation of Immune Cell Activity

**Figure 6:**
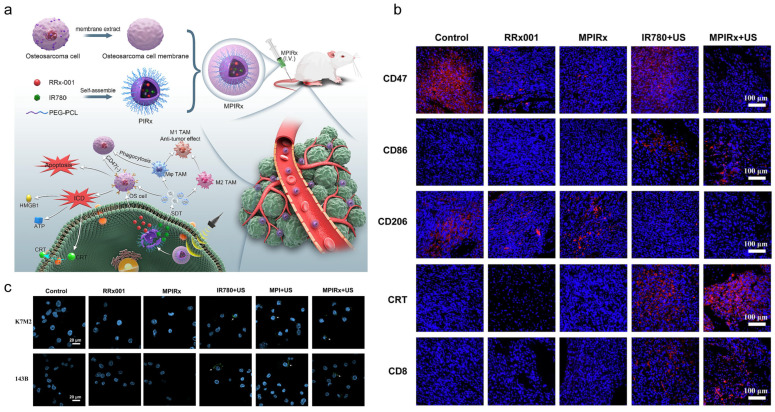
(**a**) Construction of MPIRx nanodrugs for CD47 immune-checkpoint/sonodynamic therapy of OS and pulmonary metastasis. (**b**) Representative immunofluorescence images of tumor sections by different treatments showing CD47, CD86, CD206, and CRT expression. (**c**) Effect of MPIRx nanodrugs on ICD of OS cells. Calreticulin on the outer surface of OS cells was imaged by LSCM. Adapted with permission from Ref. [32]. Copyright 2023 Elsevier.

Nanomaterials can be engineered to specifically target immune cells within the OS tumor microenvironment (TME), including macrophages, dendritic cells, and T lymphocytes. For instance, aluminum-based nanosheets (nAl) enable co-delivery of chemotherapeutic agents and immunomodulators, driving macrophage polarization from pro-tumorigenic M2 to antitumor M1 phenotypes to amplify antitumor immunity [30]. Additionally, activating CD8+ T-cell infiltration through nanoplatforms can reprogram immunologically “cold” tumors into “hot” phenotypes [31]. Immunomodulatory approaches are exemplified by MPIRx nanomedicine, which co-delivers IR780 and RRx-001 in PEG-PCL nanocompartments to promote macrophage phagocytosis and M1 polarization via CD47 inhibition, offering a novel strategy for macrophage-associated immunotherapy (Figure 6) [32].

#### 2.2.2. Remodeling the Immunosuppressive Microenvironment

The OS TME exhibits profound immunosuppression, characterized by elevated immune checkpoint molecules (e.g., PD-1/PD-L1) and inhibitory cells (e.g., regulatory T cells) [33]. Nanomaterials counteract immune evasion by delivering checkpoint inhibitors or disrupting immunosuppressive pathways such as Wnt/β-catenin signaling [34,35]. Select nanocarriers induce immunogenic cell death (ICD) in tumor cells, triggering damage-associated molecular pattern (DAMP) release to enhance antigen presentation and adaptive immune activation [36,37]. Lipid nanoparticles or biomimetic carriers deliver tumor-specific antigens to elicit antigen-specific T-cell responses or induce immune tolerance (for autoimmune applications). Bioinspired nanovehicles mimicking cellular membrane structures prolong circulation and enable lymph node-targeted antigen delivery, bolstering antitumor immunological memory [17].

### 2.3. Catalytic Properties

#### 2.3.1. Enzyme-Mimetic Catalytic Activity

Nanomaterials can mimic natural enzymes such as peroxidase (POD) and catalase (CAT), catalyzing TME-specific substrates (e.g., H_2_O_2_, lactate) to generate cytotoxic ROS like hydroxyl radicals (•OH). For example, CuO@CeO_2_ heterojunction nanocatalysts synergize photothermal effects with chemodynamic therapy (CDT) under near-infrared (NIR) irradiation, enhancing H_2_O_2_ decomposition efficiency and ROS production [38,39]. RhRu/Ti_3_C_2_Tₓ nanozymes exhibit dual POD-like and CAT-like activities, generating •OH while decomposing H_2_O_2_ into O_2_ to alleviate tumor hypoxia and amplify photodynamic therapy (PDT) efficacy [40].

#### 2.3.2. Cascade Catalytic Reactions

Nanomaterials can initiate multi-step catalytic reactions in response to tumor-specific conditions (e.g., high lactate, low pH, elevated GSH). Lactate-responsive CuO@CeO_2_ nanosystems activate cascade catalysis to deplete glutathione (GSH) and produce ROS, inducing apoptosis and ferroptosis [41]. Tong et al. bone-targeted silica nanoreactor achieved concentrated OS deposition and prolonged retention, utilizing laser-activated chlorin e6 for dual ROS generation and controlled DOX/doxycycline release, where doxycycline amplified oxidative stress to potentiate chemotherapy [42].

#### 2.3.3. Exogenous Field-Enhanced Catalysis

**Figure 7:**
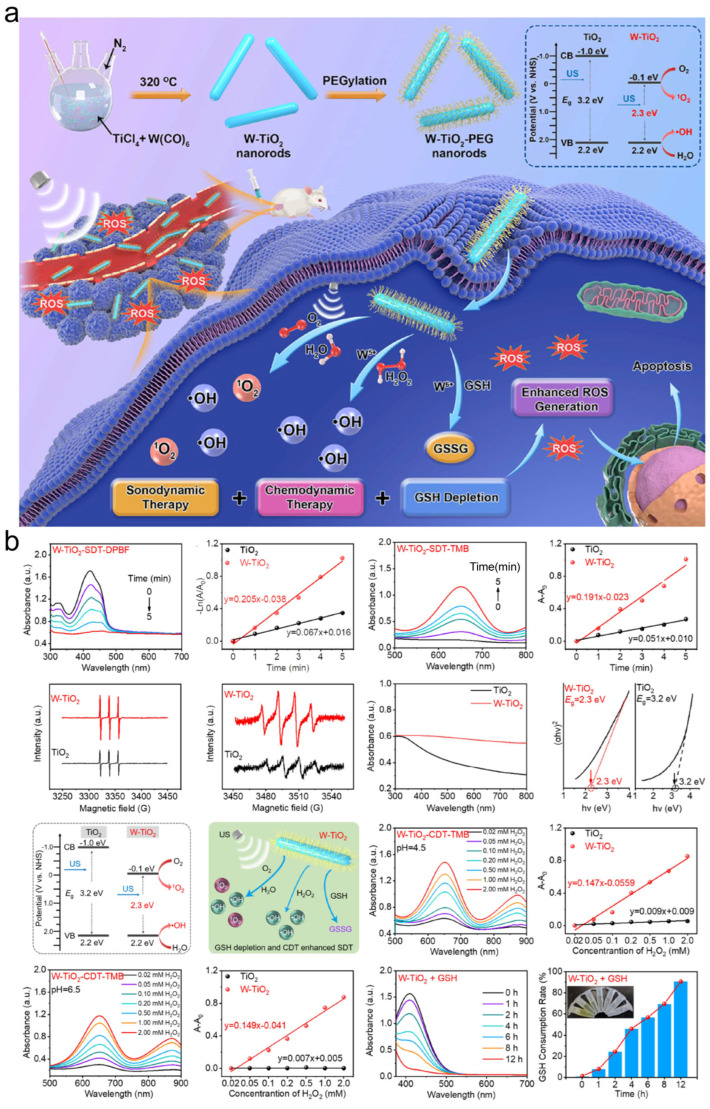
(**a**) Overall scheme of the preparation procedure for ultrafine W-TiO_2_ nanorods with TME-regulating ability for CDT-enhanced SDT. (**b**) Schematic illustration of the enhanced ROS generation based on integrating SDT, CDT, and GSH depletion via W-TiO_2_ nanorods. Adapted with permission from Ref. [43]. Copyright 2021 American Chemical Society.

External stimuli (e.g., light, heat, ultrasound) further amplify catalytic efficiency: NIR-II-driven FeS@CP NPs accelerate •OH generation and GSH depletion under irradiation, enabling synergistic photothermal/chemodynamic therapy [39]. Geng et al. synthesized W-doped TiO_2_ nanorods via high-temperature solution methods, where W^5+^/W^6+^ doping enhanced ROS generation for ultrasound-activated tumor ablation with rapid renal clearance (Figure 7) [43].

### 2.4. Thermal Effect

Thee thermal effects of nanomaterials refer to their capacity to absorb external energy (e.g., light, magnetic fields, electricity) and convert it into thermal energy.

#### 2.4.1. Photothermal Effects

**Figure 8:**
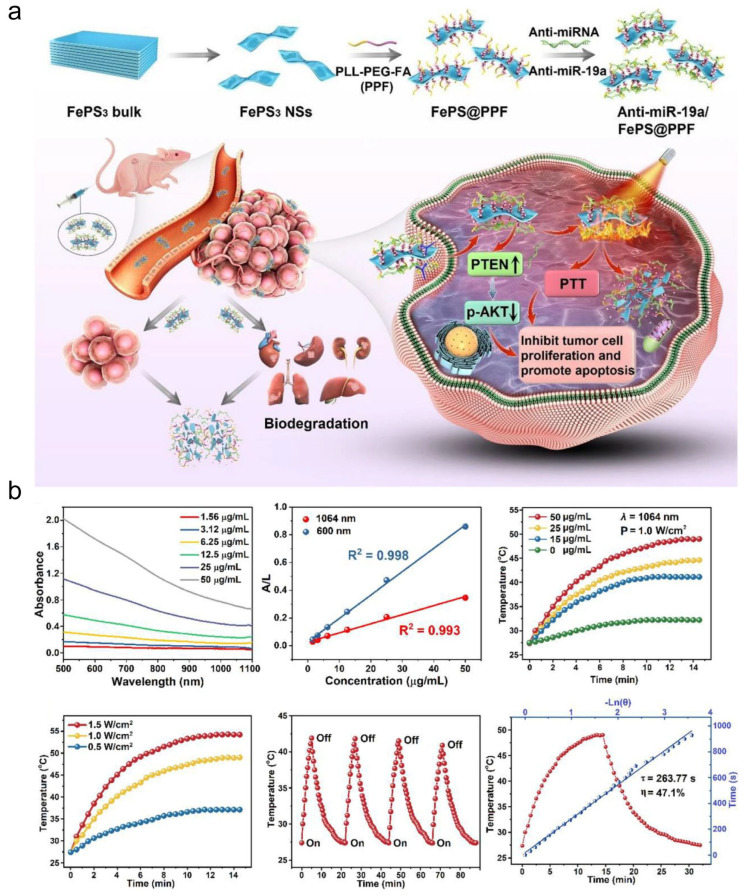
(**a**) The fabrication of anti-miRNA-loaded FePS3 nanoplatform (anti-miR-19a/FePS@PPF) for combination treatment of OS. (**b**) NIR-II photothermal effects of FePS@PPF. Adapted from Ref. [46].

Under near-infrared (NIR) excitation, nanomaterials such as polydopamine, carbon-based materials, and metal sulfides convert light energy into localized hyperthermia (42–50 °C), inducing direct tumor cell ablation [44,45]. For instance, FePS_3_ nanosheets enable efficient OS ablation through photothermal conversion (Figure 8) [46].

#### 2.4.2. Magnetothermal Effects

Magnetic nanomaterials (e.g., iron oxides) generate eddy current-induced heat in alternating magnetic fields, facilitating temperature-modulated drug release or synergistic immunotherapy [47,48]. Notably, magnesium-based materials exhibit enhanced antitumor efficacy via thermal effects under low-intensity alternating magnetic fields [47]. Under electric field stimulation, nanomaterials generate therapeutic heat levels to enhance cellular endocytosis or drug delivery efficiency [49].

### 2.5. Tissue Engineering

#### 2.5.1. Bioactivity Regulation

Nanomaterials can be engineered as multifunctional systems for precise delivery of immunomodulators, growth factors, or gene therapies. Surface modifications enable targeted release mechanisms, amplifying localized bioactivity while minimizing systemic toxicity [50]. Nanocarriers (e.g., mesoporous silica, metal-based materials) encapsulate natural agents like quercetin, achieving tumor microenvironment-responsive release via pH or ROS triggers to enhance therapeutic precision [51].

#### 2.5.2. Tissue Regeneration and Biomimetic Support

**Figure 9:**
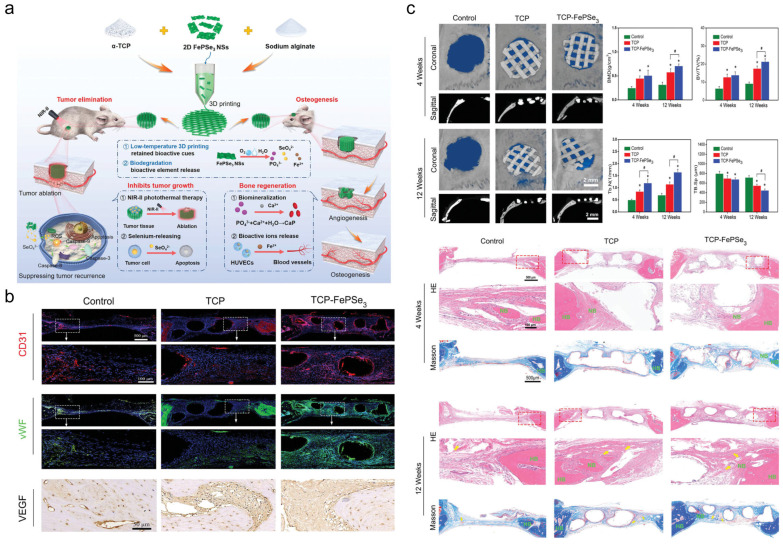
(**a**) Schematic illustration of the 3D-printed TCP-FePSe3 scaffold as an all-in-one platform for bone regeneration and postsurgical suppression of OS recurrence. (**b**) Bone defect repair in vivo after scaffold implantation assessed by immunofluorescence and immunohistochemical labeling. (**c**) In vivo osteogenesis performance of TCP-FePSe3 scaffolds. (Yellow arrows as blood vessel, HB indicated as host bone, NB as new bone). (*n* = 5), * *p* < 0.05 when comparing TCP/TCP-FePSe3 scaffolds to the control group; # *p* < 0.05 in comparison of TCP-FePSe3 scaffolds to the TCP group.). Adapted with permission from Ref. [52]. Copyright 2023 John Wiley and Sons.

Nanomaterials (e.g., hydroxyapatite, calcium phosphate composites) mimic native bone matrix composition and microstructure, promoting osteoblast adhesion, proliferation, and differentiation to accelerate bone defect repair [52,53]. As shown in Figure 9, Xu et al. designed a low-temperature 3D-printed tricalcium phosphate (TCP)-FePSe3 scaffold integrating FePSe3 nanosheets into α-TCP matrices, combining OS therapy and bone regeneration. This system enables efficient photothermal tumor ablation while releasing therapeutic ions: Se induces tumor apoptosis, while Fe/Ca/P promote angiogenesis and bone repair. The low-temperature fabrication preserves bioactivity and enables customized anatomical fitting for complex bone defects [48]. For example, neodymium/manganese co-doped whitlockite nanoparticles within alginate hydrogels simultaneously exhibit osteoinductive and antitumor properties [54]. Multifunctional scaffolds (e.g., drug-loaded silk fibroin/nano-hydroxyapatite fibers) eradicate residual tumor cells while providing structural support and bioactive cues for bone regeneration. This dual functionality significantly reduces postoperative recurrence and restores bone integrity [55].

## 3. Nanomaterials for Treatment of OS

### 3.1. Nanovesicle

**Figure 10:**
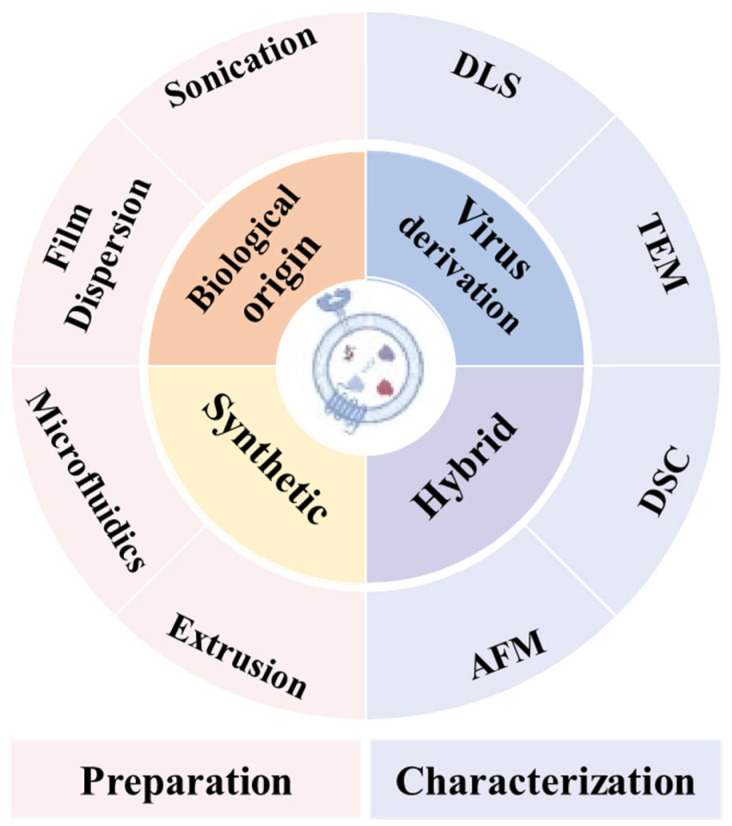
Classification of nanovesicles and methods used for their preparation and characterization. Reprinted from Ref. [61]. (DLS: Dynamic Light Scattering; TEM: Transmission Electron Microscopy; DSC: Differential Scanning Calorimetry; AFM: Atomic Force Microscopy).

Nanovesicle materials (Table 1) represent a class of hollow or semi-hollow nanoscale carriers characterized by customizable functionality through molecular design. As shown in Figure 10, nanovesicles play a pivotal role in OS therapeutics and can be categorized into four classes: biologically derived, virus-derived, synthetic, and hybrid nanovesicles. Each variant demonstrates distinct functional characteristics: (1) Biologically derived nanovesicles originate from natural cellular sources including tumor cells and mesenchymal stem cells. These structures preserve native membrane composition and architecture while transporting diverse biomolecules. Their advantages include superior biocompatibility with minimal immunogenicity, inherent tumor-targeting capabilities through surface biomarkers, and multifactorial therapeutic effects via combined delivery of bioactive components. However, limitations encompass low production yield, compositional heterogeneity, and compromised in vivo stability [56]. (2) Virus-derived nanovesicles are engineered through pathogenic gene deletion while retaining viral transduction capacity. These vectors enable efficient delivery of therapeutic cargo (drugs/genes) to tumor cells, with modifiable tropism for targeted action. Challenges include residual immunogenicity potentially triggering adverse immune responses, latent pathogenicity risks, and complex manufacturing processes requiring substantial technical expertise [57]. (3) Synthetic nanovesicles, exemplified by liposomal and polymeric systems, feature programmable composition and architecture through controlled fabrication. Key advantages include precise engineering of physicochemical properties, enhanced storage stability, high drug-loading capacity, and capacity for combination therapies. Drawbacks involve reduced biological recognition requiring artificial targeting ligands and potential cytotoxicity from synthetic materials [58]. (4) Hybrid nanovesicles integrate biological and synthetic components to synergize therapeutic advantages. This chimeric design enables performance optimization through material hybridization while addressing individual system limitations [59]. Current technical barriers include intricate manufacturing protocols, stringent quality control requirements, and challenges in batch-to-batch reproducibility. Current nanovesicle systems for OS therapy primarily include liposomes, exosomes, virus-like particles, and cell membrane vesicles [60].

**Table 1:** Representative paradigms of biovesicular nanomaterials for the treatment of OS.

Nanomaterial	Cell Line	Functional Properties	Tumor Model	Inhibition Rate	Innovation Points	Ref.
EXO-RIF	143B, MG63	Rifampicin delivery	Xenograft nude mice	67.3%	BMSC exosomes, Drp1 agonist	[62]
EM-Dox	143B	DOX delivery	carcinoma in situ	82.9%	Exosome mimetics (EMs)	[63]
Exo-Dox	MG63, 143B	DOX nanocarrier	Xenograft nude mouse	83.3%	SDF1-CXCR4 targeted therapy	[64]
BT-Exo-CAP	MG63, 143B	Carrying CAP	carcinoma in situ	75%	Bone targeting, ferroptosis	[65]
DAELNs	HOS, MG63	OS-targeted agents	Xenograft model	70%	Dipsacus asperoides-derived P38/JNK signaling pathway	[66]
YSA-SPION-MV/MTX	MG63, 143B	MTX delivery	Orthotopic model	significantly inhibited	EphA2 magnetic targeting	[67]
ALN-LWMH-DOX-Lip	K7M2	DOX delivery	Orthotopic model	79.2%	Bone targeting, anti-metastasis	[68]
HA-DOPE@Lips/HNK	143B	Honokiol delivery	Xenograft model	63.7%	Hyaluronic acid HNK	[69]
FA-Res/Lps	143B	Resveratrol delivery	Metastasis models	82.9%	Lung metastasis JAK2/STAT3 pathway	[70]

#### 3.1.1. Liposomes

Liposomes, featuring phospholipid bilayer structures mimicking biological membranes, demonstrate superior biocompatibility and low toxicity. These nanostructures have emerged as prominent drug delivery systems in OS management [71]. To address platinum-based chemotherapy limitations, Yan et al. developed liposome-encapsulated Pt prodrugs (Lipo-OXA-ALN), enhancing tumor-specific accumulation while minimizing systemic toxicity [72]. Wei’s team employed liposomal miRNA delivery to reverse cisplatin resistance by targeting the METTL3/LINC00520 axis through glycolytic pathway modulation [26]. Surface engineering through polymeric or protein modifications enhances liposomal performance. Wu et al. constructed a dual—functional liposome system modified with alendronate (ALN) and low-molecular-weight heparin (LMWH) for targeted DOX delivery to bone tumors. ALN targets bone tissue and combats osteoporosis, while LMWH prolongs circulation and suppresses metastasis. Their synergistic action enhances drug enrichment and reduces side effects [68]. Zhang et al. developed a novel HA-DOPE@Lips/HNK liposome carrier. It uses hyaluronic acid (HA) to bind to CD44 receptors, enabling active targeting of OS. This approach addresses the challenges of honokiol (HNK)’s poor water solubility and lack of targeting [69]. Zhu et al. developed FA-Res/Lps for resveratrol delivery. FA binds to FR on tumor cells for active targeting, solving resveratrol’s solubility and bioavailability issues. This enhances tumor enrichment and antitumor efficacy. Mechanistic studies show it acts via JAK2/STAT3 inhibition, revealing a new molecular target [70]. Turánek et al. demonstrated that HPMA copolymer-modified liposomes exhibit prolonged circulation without immunogenicity compared to PEGylated counterparts [73]. SPI or 7S/11S globulin coatings significantly improve colloidal stability [74,75]. Chen’s team designed cationic liposomes with enhanced membrane interaction for improved vaccine adjuvant efficacy [76]. Multifunctional liposomal systems enable combination therapies. Song et al. developed catalytic liposomes (Au/PDA/HRP@DLP) integrating SDT and photothermal therapy (PTT) for dual tumor ablation and immune activation [77]. Zhang’s T-APR-246-loaded liposomes effectively remodeled the immunosuppressive microenvironment [78]. Despite promising preclinical results in toxicity reduction (e.g., DOX cardiotoxicity mitigation), clinical translation requires resolution of manufacturing scalability and long-term safety challenges [79]. Current research focuses on developing stimulus-responsive or actively targeted liposomes to overcome tumor penetration limitations [17,80].

#### 3.1.2. Exosomes

Exosomes, naturally secreted nanoscale vesicles, serve as critical intercellular communicators by transferring biomolecules (proteins, nucleic acids, lipids) between cells. These membrane-bound structures are ubiquitously present in bodily fluids, including blood, urine, and saliva. Han et al. identified elevated plasma exosomal protein markers (CD63, VIM, EpCAM) in OS patients, suggesting diagnostic potential [81]. Almeida’s team engineered tumor-derived exosomes as PET imaging probes for detecting occult pulmonary metastases [82]. While BMSC-derived exosomes offer biocompatible drug delivery platforms, their intrinsic targeting limitations risk off-site effects [65,67]. Wei et al. leveraged MSC-derived exosomes’ intrinsic OS targeting via SDF1-CXCR4 axis chemotaxis to achieve tumor-specific DOX accumulation. The Exo-Dox system significantly mitigated DOX-induced cardiotoxicity while overcoming the dose-limiting cardiotoxicity in clinical applications [64]. Ji et al. engineered bone-targeting peptide (SDSSD)-modified BMSC exosomes (BT-EXO-CAP) delivering capreomycin, achieving synchronized bone/homing targeting for enhanced drug delivery. The system induced OS ferroptosis via ROS/Fe^2^⁺/lipid peroxidation cascades, mechanistically involving Keap1/Nrf2/GPX4 axis modulation [65]. Chen et al. developed exosomes from bone marrow mesenchymal stem cells to deliver rifampicin nanodrugs, discovering this anti-tuberculosis agent’s novel anti-OS effect. Their mechanistic study revealed rifampicin activates Drp1 protein, inducing excessive mitochondrial fission and triggering tumor cell apoptosis [62]. Du et al. enhanced OS targeting through NGR peptide-modified CAF exosomes, delivering circ_0004872-encoded peptides to induce autophagy-dependent ferroptosis [83]. Jiang’s group demonstrated exosomal miR-4443-mediated ferroptosis via ATF3 regulation [84]. Liu’s research revealed tumor-derived migrasomes as microenvironment modulators promoting metastasis [85]. Huang et al. identified tumor-suppressive effects of exosomal lncRNA MEG3 through oncogenic pathway inhibition [86]. Engineered exosomes demonstrate immunotherapeutic potential through inflammatory mediator delivery. Sun et al. characterized DC-derived exosomes mediating PD-L1-dependent immunosuppression [87]. Wang’s team proposed serum exosomal PD-L1 as both prognostic biomarker and therapeutic target for checkpoint inhibitor optimization [88]. Wang et al. developed bone marrow stem cell-derived exosome mimetics (EMs) via sequential extrusion for DOX delivery, yielding 20-fold more EMs than natural exosomes while maintaining tumor targeting. Using ammonium sulfate gradients, they achieved efficient DOX encapsulation (0.8 mg/mL). The EM-Dox system exhibited pH-responsive release, preferentially liberating drugs in acidic tumor microenvironments (pH 5.5) [63]. Lu et al. isolated exosome-like nanoparticles from Dipsacus asperoides, revealing their first-reported anti-OS activity through p38/JNK pathway activation. These plant-derived vesicles demonstrate precise targeting and low toxicity, advancing phytogenic nanomedicine applications [66].

#### 3.1.3. Virus-Like Particles

Virus-like particles (VLPs), synthetic nanoscale vesicles mimicking viral morphology without containing genetic material, combine non-infectious properties with preserved antigenic surface features capable of eliciting immune responses. VLP-based strategies in OS management focus on two key applications: precision nanocarrier systems and immunotherapeutic enhancement. These engineered structures demonstrate exceptional drug/gene delivery potential through their inherent biocompatibility and nanoscale architecture. Suffian et al. developed VLP-encapsulated CRISPR-based gene-editing systems for modulating metastasis-related pathways [89]. The immunostimulatory capacity of VLPs enables tumor-specific antigen presentation. Yin’s team engineered DC-targeting VLPs displaying OS antigens to potentiate T-cell activation [90]. Alvaro et al. demonstrated synergistic antitumor effects through VLP/oncolytic virus combinations, achieving enhanced tumor lysis via dual mechanisms [91].

### 3.2. Polymer

#### 3.2.1. Poly (Lactic-c–o-Glycolic Acid)

Poly (Lactic-co-Glycolic Acid) (PLGA) has emerged as a promising platform for OS therapy due to its biocompatibility, biodegradability, and controlled drug release capabilities. Recent advances demonstrate enhanced therapeutic efficacy through innovative nanoformulations (Table 2): Cai et al. created PTX-PLGA@[143B-RAW] nanoparticles by ultrasound-assisted paclitaxel loading on PLGA cores wrapped with hybrid OS/macrophage membranes. This dual-targeting system combines homologous tumor recognition and inflammatory chemotaxis, enhancing drug delivery. In vitro tests revealed pH-responsive release (90% at pH 5.3 vs. 70% neutral) and efficient tumor cell uptake, offering a biomimetic strategy for precise OS therapy [92]. Alsulays et al. developed chitosan-coated IVO-CS-PLGA-NPs to improve pharmacokinetic stability and tumor targeting of ivosidenib [93]. Biomimetic engineering approaches, exemplified by Wang’s IR780@PLGA@HM platform utilizing cancer cell membrane coating, enable microenvironment-specific targeting and immunomodulation [94]. Multimodal therapeutic strategies achieve synergistic effects through rational material design. Huang et al. combined DOX-loaded PLGA nanoparticles with polydopamine for NIR-triggered chemo-photothermal synergy [16], while Crisafulli’s layer-by-layer chitosan-PLGA system co-delivers miRNA-34a with chemotherapeutics to overcome drug resistance [95]. Advanced formulations like Wang’s MnO/Fe@PLGA nanoparticles leverage metal ion-mediated ROS generation and macrophage polarization to prevent postoperative recurrence. PLGA-based platforms also address antimicrobial challenges in cancer therapy through photothermally enhanced antibacterial peptide delivery. Furthermore, Sapino’s P-gp inhibitor-loaded nanoparticles demonstrate improved chemosensitivity by blocking drug efflux mechanisms, highlighting PLGA’s potential in overcoming conventional chemotherapy limitations. Li et al. designed a dual-responsive (redox/pH) mPEG-PaLA copolymer nanocarrier for co-delivering PTX and DOX in OS treatment. In K7 models, NP-PTX-DOX exhibited superior tumor suppression over single-drug and free-drug groups, confirming synergistic therapeutic efficacy [96]. Wang et al. developed MH-PLGA-IR780 NPs, a homologous-targeted nanoplatform using HOS membrane-coated PLGA nanoparticles encapsulating IR780. This system integrates dual-modality imaging (photoacoustic/fluorescence) with NIR-triggered photodynamic therapy, inducing concurrent apoptosis and ferroptosis in OS. Mechanistically, ROS accumulation triggers mitochondrial apoptosis and NCOA4-mediated ferritinophagy while suppressing GPX4, causing lethal lipid peroxidation. The HOS membrane coating enables immune evasion and deep tumor penetration, overcoming conventional nanoparticle limitations in OS therapy [97].

#### 3.2.2. Polydopamine

Polydopamine (PDA) nanomaterials, formed via oxidative self-polymerization of dopamine monomers under alkaline conditions, demonstrate significant potential in OS therapy due to their photothermal conversion and radical scavenging capabilities. Liu et al. developed a PDA-MOF-E-M nanocomposite integrating tumor-targeting ligands, anti-PD-1/PD-L1 immune-checkpoint inhibitors, and near-infrared (NIR) photothermal effects for multimodal therapy. This combination significantly enhanced tumor cell eradication through photothermal/chemotherapy synergy while suppressing bone destruction progression in vivo [25]. Ma’s team engineered PDA@MnS nanostructures that induce localized hyperthermia and catalyze ROS generation, establishing a dual PTT-CDT therapeutic mechanism. This strategy achieved enhanced antitumor efficacy with reduced systemic toxicity through low-dose chemotherapeutic synergy [110]. PDA-based platforms effectively deliver immunoadjuvants (e.g., CpG oligonucleotides) or chemotherapeutics, triggering immunogenic cell death (ICD) to activate T-cell responses and potentiate anti-PD-1 therapy [111]. Furthermore, Li et al. demonstrated that PDA@HA coatings mitigate oxidative stress in tumor microenvironments via ROS scavenging, effectively inhibiting osteoclast activation and bone degradation [112].

#### 3.2.3. Hydrogel

Polymer hydrogels, three-dimensional networks formed through crosslinked polymer chains, exhibit exceptional water absorption and retention capacities. Characterized by high hydration, tunable mechanics, and biocompatibility, these materials demonstrate dual therapeutic and regenerative potential in OS management. Classified by origin, they encompass natural (e.g., collagen, alginate) and synthetic variants (e.g., poly(*N*-isopropylacrylamide), polyvinyl alcohol). Innovative in situ crosslinking systems like MOG hydrogel—comprising MgO nanoparticles and dopamine-conjugated gelatin—effectively fill post-resection defects while delivering localized electrostimulation via mild ultrasound. This approach achieved 67.6% tumor suppression in tibial models while stimulating bone regeneration [113,114]. Si et al. created an injectable thermoresponsive PLGA-PEG-PLGA hydrogel for localized co-delivery of DOX and CDDP. The material’s temperature-dependent phase transition enables sustained drug release at tumor sites, enhancing local drug concentration by 2.7-fold while reducing systemic exposure by 58–63%, with 96 h retention and synergistic cytotoxicity [107]. Freeman et al. demonstrated that localized miR-29b delivery concurrently inhibits OS growth and restores bone homeostasis. They engineered an injectable hyaluronic acid (HA)-based hydrogel enabling sustained release of miR-29b-encapsulated poly(β-amino ester) (pBAE) nanoparticles, achieving precision-controlled miRNA delivery as an adjunct to conventional chemotherapy during tumor resection [108]. Wang et al. engineered an injectable hydrogel for sequential Nox4i/L-Dox delivery. Nox4i inhibits CAF activation to reverse T-cell exclusion, while L-Dox induces immunogenic cell death, amplifying antitumor immunity. Clinical data link Nox4 overexpression to poor OS prognosis, validating this sequential therapy [109]. Luo’s FePSe nanocomposite hydrogel synergizes photothermal ablation with controlled chemotherapeutic release, demonstrating 35.6-fold tumor volume reduction and reduced recurrence [46]. Xie’s Mg^2^⁺/nHA-incorporated PH-GBS@CCP hydrogel sustains bioactive ion release for critical-size defect repair [115]. Electrically conductive variants like polyacrylic acid-based composites promote osteoblast differentiation while inhibiting tumor proliferation through electrostimulation [116,117]. Significantly, hydrogels modulate tumor immune microenvironments by reprogramming CAFs via TGF-β regulation, enhancing T-cell infiltration and suppressing metastasis [109,118]. Combinatorial systems co-delivering αPD-L1 antibodies and Hedgehog inhibitors demonstrate enhanced checkpoint blockade efficacy [119,120]. Clinically transformative applications include 3D-bioprinted models incorporating extracellular matrix mimics for personalized drug screening [121]. Key challenges include (1) mechanical optimization via nanofiber reinforcement or supramolecular crosslinking to balance flexibility and strength [122,123]; (2) overcoming chemoresistance through CRISPR-identified targets like RFWD3 in gene-silencing hydrogels [124]; and (3) infection control using antimicrobial formulations integrating silver nanoparticles or antibiotics [125].

### 3.3. Inorganic Nonmetal

#### 3.3.1. Calcium-Based Nanomaterials

Calcium-based nanomaterials (e.g., hydroxyapatite, calcium phosphate) demonstrate unique advantages in OS treatment due to their inherent biocompatibility and osseointegration capacity. These materials degrade into metabolically compatible Ca^2^⁺ and PO_4_^3−^ ions, offering superior safety over conventional metallic or polymeric carriers. Major calcium-based variants (Table 3) include hydroxyapatite (HAp), tricalcium phosphate (TCP), calcium carbonate (CaCO_3_), calcium peroxide (CaO_2_), and perovskite-type nanomaterials.

As the principal inorganic component of bone, nano-hydroxyapatite exhibits exceptional biocompatibility and osteoconductivity. Its nanoparticulate form enhances osteoblast activity while providing high surface area for drug/growth factor loading. Wang et al. first demonstrated nano-HAPs concentration-dependently inhibit OS OS-732 cell viability, migration, and invasion via FAK/PI3K/Akt downregulation. Intratumoral injection suppressed tumor growth comparably to combined implantation [126]. Liu et al. developed a hydroxyapatite/BSA/paclitaxel ternary nanoparticle system for postoperative OS therapy, achieving 32.17% drug loading with sustained PTX/calcium release, low cytotoxicity, and enhanced osteogenic differentiation for bone repair [127]. Liu et al. developed HA nanoparticle/microparticle carriers for doxorubicin delivery, achieving pH-dependent drug release via OS cellular internalization. Mitochondrial-targeted DOX induced apoptosis through metabolic disruption, while the dual-particle system enhanced safety and sustained release [128]. Wu et al. demonstrated morphology/size-dependent HANPs selectively inhibit OS cells with low toxicity. Rod/needle-shaped variants showed optimal efficacy via calcium/ROS elevation and mitochondrial apoptosis while inducing immunogenic cell death. HANPs trigger immunogenic cell death by releasing DAMPs (CRT, ATP, HMGB1) to activate innate immunity and polarize macrophages toward the M1 phenotype while enhancing CD8+ T-cell infiltration, with the highest-aspect-ratio HANPs-3 exhibiting optimal immunomodulatory and antitumor effects [129,130]. Xie et al. developed 3D-printed polylactic acid scaffolds with surface-modified HAp (PH-GBS@CCP), achieving simultaneous bone regeneration and localized cisplatin release through hydrogel integration [115]. HAp’s tumor-suppressive mechanism involves calcium-mediated apoptosis induction via dense mineralization layers on OS cells, demonstrating superior efficacy to DOX in vitro [29]. Metal-doped HAp nanoparticles (ZnHAU, FeHAU) enable pH-responsive drug release through hydrogen bonding and metal–ligand interactions, minimizing systemic toxicity [144,145]. Antimicrobial HAp composites incorporating MgO/FeO nanosheets or antibacterial peptides effectively inhibit biofilm formation while promoting osseointegration [146,147].

Calcium phosphate nanomaterials enable tumor–bone dual therapy through functional composites. FePSe-integrated cryogenic-3D-printed scaffolds achieve synergistic osteogenesis and tumor suppression [52]. Germanium–selenium co-doped TCP bioceramic regulate neurogenesis and angiogenesis for combined tumor treatment and bone repair [148]. Tumor microenvironment-responsive calcium phosphonate nanoagents enable sustained drug release and immune activation, suppressing pulmonary metastasis through dendritic cell maturation and CD8⁺ T-cell recruitment [149]. Calcium phosphate nanoparticles disrupt tumor calcium homeostasis via mitochondrial dysfunction, while zinc-doped variants exhibit pH-dependent antitumor activity [150,151].

Calcium carbonate nanomaterials induce tumor-specific calcium overload through acidic microenvironment-responsive degradation. Gadolinium-stabilized amorphous calcium carbonate (ACC) achieves Magnetic Resonance Imaging (MRI)-trackable theranostics with enhanced stability [152,153]. Hyaluronic acid-coated ACC nanoparticles (H-CNP@pGMD) trigger pyroptosis and antitumor immunity via lysosomal degradation-activated gene delivery [154]. Organic solvent-stabilized ACC nanospheres show promise for controlled drug delivery [155,156]. Wang et al. developed europium-doped calcium fluoride (CaF_2_:Eu) nanoparticles for OS radiotherapy enhancement, demonstrating selective cytotoxicity, migration suppression, and X-ray sensitization via amplified radiation interactions to reduce proliferation and metastasis [132].

#### 3.3.2. Carbon-Based Nanomaterials

Carbon-based nanomaterials, composed primarily of carbon atoms at nanoscale dimensions, have found extensive applications in oncotherapy. Representative materials include graphene, carbon nanotubes (CNTs), carbon dots (CDs), fullerene (C60), mesoporous carbon nanospheres (MCNs), and nanodiamonds (NDs).

Graphene derivatives, particularly graphene oxide nanosheets, exhibit exceptional surface-to-volume ratios, mechanical strength, and functionalizable surfaces, serving as efficient nanocarriers for chemotherapeutic agents like DOX through physical adsorption or covalent conjugation [157,158]. These platforms enable multimodal OS therapy by integrating targeting ligands (e.g., hyaluronic acid) with combinatorial chemo-photothermal-photodynamic approaches. Near-infrared (NIR)-responsive graphene composites generate localized hyperthermia and reactive oxygen species (ROS) under irradiation, achieving synergistic tumor ablation [159]. Notably, graphene-modified bone scaffolds regulate osteogenic differentiation via RUNX2 and OCN expression, facilitating post-resection bone regeneration [160,161]. Optimized parameters (<20 mg/kg dose, 50–1000 nm size, PEGylation) enhance biocompatibility while mitigating inflammatory responses [162]. Huang et al. fabricated PEG-GO-FA/ICG nanoparticles for chemo-photodynamic therapy. They enhanced photodynamic efficacy by inhibiting MTH1, inducing apoptosis via ER stress and JNK/p53/p21 pathways, with autophagy offering protection [139].

Carbon dots (CDs) enable dual diagnostic/therapeutic functionality through tunable fluorescence and surface engineering. NIR-emitting CDs demonstrate concurrent photothermal therapy and chemodynamic effects via Fenton-like •OH generation [163]. Their sub-10 nm dimensions enhance tumor penetration and multidrug resistance reversal. Mn-doped CDs achieve MRI-fluorescence bimodal imaging while catalyzing ROS production in tumor microenvironments [164]. Advanced applications include boron neutron capture therapy via B-enriched CDs [165] and antibacterial/osteogenic 3D hydrogels for infected bone defects [166]. Their favorable biosafety profile (<100 μg/mL cytotoxicity) and biodegradability support clinical translation [167,168]. Consoli et al. prepared phenol/carboxyl-containing Carbon-BNQDs via a DMF-based one-step method. These water-dispersible dots have pH-dependent fluorescence, high biocompatibility, and photothermal efficiency (60.7% in solution, 90% in solid state) [142].

Carbon nanotubes (CNTs), categorized as single-walled (SWCNTs) or multi-walled (MWCNTs), exhibit unique advantages in modulating tumor immune microenvironments. MWCNTs disrupt cytoskeletal dynamics, mimicking microtubule-targeting agents like paclitaxel [168]. Magnetic CNTs (mCNTs) implement mechanotherapeutic strategies by exerting mechanical stress to rupture tumor cells under magnetic guidance [169,170]. Gisbert-Garzarán et al. engineered pH-responsive CMK-3/1 mesoporous carbon nanoparticles coated with self-immolative polyurethane, demonstrating enhanced drug loading over MSNs and precise pH-dependent release validated by in vitro/vivo biocompatibility studies [138].

#### 3.3.3. Silicon-Based Nanomaterials

Silicon-based nanomaterials constitute a diverse class of nanostructures with silicon as the core element, including silica nanoparticles (SiO_2_ NPs), mesoporous silica nanoparticles (MSNs), bioactive glass (BG), silicon quantum dots (Si QDs), and silicon nanowires (SiNWs).

Mesoporous silica nanoparticles (MSNs), characterized by ordered pore structures and tunable surface chemistry, serve as versatile platforms for OS therapy. He et al. engineered three selenium-loaded mesoporous silica nanoparticles (surface/pore-loaded, matrix-doped, core-loaded) demonstrating rapid controllable selenium release, enhanced under reductants, with Se-doped variants showing selective Saos-2 cytotoxicity and minimal hFOB 1.19 toxicity [134]. Ye et al. designed GSH/pH-responsive organic/inorganic hybrid MCD nanoparticles exhibiting photothermal/antibacterial activity, which combat OS via cuproptosis/ROS synergy while promoting osteogenic differentiation, amplified by NIR-II irradiation [135]. Metal co-doped MSNs (e.g., Cu&Ce-MSNs) enable NIR-II fluorescence/MRI dual-modal imaging while amplifying ROS generation and metabolic interference for enhanced tumor suppression [171]. Aptamer/antibody-functionalized MSNs improve tumor targeting via CD47 recognition, enhancing phagocytosis and drug accumulation [172]. The hierarchical porosity facilitates spatiotemporally controlled release of chemo-gene combination therapies (e.g., temozolomide/siHDGF co-delivery), effectively overcoming drug resistance [173]. Immunotherapeutic applications exploit mannose-modified MSNs to activate DC-mediated T-cell responses, significantly reducing pulmonary metastasis [174]. Lanthanide-incorporated MSNs (Eu/Gd-MSNs) provide real-time therapeutic monitoring through bimodal imaging [175]. Stimulus-responsive MSNs with pH/ROS-triggered drug release mechanisms enhance therapeutic precision [176]. Surface engineering strategies (PEGylation, Mn-O bond integration) address biodegradation concerns while maintaining biocompatibility [177,178].

Silicon nanowires (SiNWs) demonstrate tumor-selective internalization advantages, with 3 × 3 µm microdevices showing preferential uptake in Saos-2 OS cells versus normal osteoblasts [179]. Pre-lithiated porous SiNWs (LipSiNs) combine lithium/silicon co-delivery with bioresorbability, enabling innovative drug/gene loading approaches [180]. Multifunctional composites like Mn-doped silicon/hydroxyapatite nanowires regulate T-cell immunity via MnSOD/AMPK pathways, concurrently suppressing inflammation and promoting osseointegration [181]. Silicene-modified bone matrices (SNS@DBM) achieve photothermal ablation and osteogenic factor release through “thermal switch” mechanisms [182].

Bioactive glass (BG), first developed by Hench in the 1970s, exhibits dynamic biointeractivity through ionic dissolution/reprecipitation processes. Typical compositions comprise SiO_2_, CaO, P_2_O_5_ with optional additions of Na_2_O, K_2_O, or therapeutic metal ions. Modern BG scaffolds integrate dual osteogenic/antitumor functions through nanocomposite designs. SeNP/MgFe-LDH-modified BG systems eradicate residual tumors via ROS/Mg^2^⁺ release while stimulating osteogenesis [183]. Strontium-doped BG modulates macrophage polarization to attenuate fibrosis and enhance angiogenesis [184]. Zn/V co-doped BG nanoparticles regulate diabetic bone healing through antioxidant delivery and immunomodulation [185]. Advanced 3D-printed CeO_2_-BG scaffolds demonstrate sequential ROS scavenging, photothermal conversion, and osteoinduction capabilities [186]. Hu et al. synthesized CTAB-templated selenium-doped mesoporous bioactive glass nanospheres (400 nm) via sol–gel methods, exhibiting high surface area (>400 m^2^/g) and pH/Se-dependent drug release with selective MG63 cytotoxicity for bone engineering applications [136].

### 3.4. Metal Based

#### 3.4.1. Noble Metal Nanoparticles

Noble metal nanoparticles (NMNs) comprise nanoscale particles of gold (Au), silver (Ag), platinum (Pt), palladium (Pd), and other noble metals. Major categories include gold nanoparticles (AuNPs), silver nanoparticles (AgNPs), platinum nanoparticles (PtNPs), and palladium nanoparticles (PdNPs), along with nanoparticles of rhodium, iridium, osmium, and related elements.

AuNPs (Table 4) exhibit diverse morphologies, including nanospheres (AuNSs), nanorods (AuNRs), nanostars (AuNSts), and hollow structures. Surface-modified AuNPs enable active tumor targeting in OS therapy. RGD-conjugated AuNPs demonstrate enhanced tumor penetration and retention in subcutaneous and pulmonary metastasis models. Mesenchymal stem cell-mediated delivery of AuNPs improves tumor targeting through cellular homing mechanisms [187]. CRISPR-Cas9 systems integrated with AuNPs achieve precise gene editing to enhance chemosensitivity in OS cells [188]. The surface-enhanced Raman scattering (SERS) properties of sub-5 nm excretable AuNPs facilitate intraoperative tumor imaging with reduced systemic retention risks [189]. AuNRs demonstrate near-infrared (NIR) photothermal conversion capabilities, with Janus-type AuNRs@MnO composites enabling multimodal photoacoustic imaging-guided chemo-photothermal therapy [190]. Biomimetic GNRs@SiO_2_@MnO platforms activate STING pathways through Mn^2^⁺ release, enhancing antitumor immunity against metastatic OS [191]. Chakraborty et al. studied the size-dependent toxicity of AuNPs on MG63 cells via a modified Frens method. Different sizes significantly affected cell viability at equal concentrations, inducing apoptosis via ROS elevation and mitochondrial membrane disruption. SERS technology was used to confirm apoptosis and monitor intracellular molecular changes [192]. Yang et al. assembled AuNR@CA hybrids via chlorogenic acid and gold nanorods. They regulated photothermal effects, enabling tumor cell inhibition and bone regeneration under near-infrared laser. Laser power was adjusted to control temperature for synergistic therapy [193]. Bu et al. developed anisotropic TOh Au@Pt nanoplatforms with edge-deposited Pt amplifying LSPR via spatially separated architecture, enabling synergistic PTT/PDT efficacy and H_2_O_2_-catalyzed oxygenation for hypoxic tumor therapy [194]. Feng et al. engineered core-shell ACZO nanoparticles (Au NRs-encapsulated Ce/Zn composites) enabling acid-triggered ROS generation, GSH depletion, and Zn^2^⁺-mediated cytotoxicity, with photothermal-enhanced CDT efficiency for synergistic antitumor efficacy [195].

AgNPs exhibit potent antimicrobial activity and SERS capabilities, though with comparatively lower biocompatibility. Dendrimer-complexed AgNPs significantly enhance antibacterial efficacy against postoperative infections [212]. Sodium alginate-coated AgNPs enable mitochondrial-targeted resveratrol delivery for improved anticancer effects [213]. Green synthesis approaches using microalgae or polysaccharides improve biocompatibility [214]. Morphological optimization (nanowires vs. nanospheres) balances bactericidal efficiency and cytotoxicity [215]. Despite progress, long-term biosafety assessments remain crucial for clinical translation [216,217]. Hu et al. developed smaller AgNPs through ultrasonic optimization, showing enhanced antitumor efficacy (outperforming cisplatin in vitro) by reprogramming glycolytic metabolism to induce apoptosis, with 68% fecal excretion within 1 week and minimal toxicity [196]. Wen et al. synthesized silver nanoparticles using tannin-rich mangrove extract via eco-friendly synthesis, demonstrating potent anti-OS activity (IC_50_ below standard thresholds) through ROS-mediated apoptosis with low cytotoxicity and therapeutic potential [197]. Cheng et al. engineered AgBiS_2_ theranostic nanoparticles integrating CT imaging (Bi/Ag-enhanced contrast) and NIR-triggered phototherapy (36.51% efficiency) with potent ROS generation and Staphylococcus aureus eradication, reducing infection risks [198].

PtNPs demonstrate catalytic versatility and chemotherapeutic potential. Acid/ROS-responsive Pt(IV) prodrug nanoparticles achieve tumor-specific drug release with reduced systemic toxicity [218,219]. Exosome-loaded ultrasmall PtNPs offer low-toxicity alternatives to conventional cisplatin [220]. Catalytic Pt-based nanomotors enhance drug diffusion in hypoxic tumor microenvironments [221]. Combinatorial strategies with RFWD3 inhibitors overcome platinum resistance through PHGDH-mediated metabolic reprogramming [128]. Immunogenic cell death induction by PtNPs synergizes with checkpoint inhibitors for enhanced therapeutic outcomes [222].

#### 3.4.2. Metal Oxide

Metal oxide nanomaterials can be categorized by metallic elements into iron-, copper-, zinc-, titanium-, and manganese-based systems, each demonstrating distinct therapeutic mechanisms in OS management.

Iron-based oxides (Fe_3_O_4_, Fe_2_O_3_) leverage superparamagnetism for targeted accumulation and Fenton reaction-mediated ROS generation. FeS@CP NPs synergize chemotherapy and photothermal therapy while promoting bone regeneration [39]. FeOOH nanosheets enhance chemodynamic therapy through Fe^2^⁺/Fe^3^⁺ cycling and GSH depletion [223]. Three-dimensionally printed scaffolds incorporating single-atom iron catalysts demonstrate tumoricidal ferroptosis induction coupled with antibacterial activity [224]. Multimodal systems like Fe-TiO nanodots combine sonodynamic and chemodynamic therapies for enhanced efficacy [225]. Zhang et al. engineered FSR-Fin56 nanoparticles (Fe_3_O_4_ core/RGD-mesoporous silica shell) for dual ferroptosis induction: Fin56-triggered GPX4 degradation and Fe^3^⁺/GSH depletion-driven Fenton catalysis. Photothermal activation amplifies ROS/LPO accumulation, enabling targeted tumor therapy [202]. Liang et al. developed DOX/Fe_3_O_4_@PMMA bone cement enabling in situ phase-transition solidification, combining magnetothermal ablation (AMF-activated) with pH/magneto-thermal dual-responsive DOX release for synergistic tumor therapy via minimally invasive delivery and precise drug control [203].

Copper-based oxides (CuO, Cu_2_O) exploit cuproptosis and Fenton-like reactions for dual therapeutic action. CuH@HA nanoparticles enable tumor-specific copper release to activate mitochondrial respiration collapse [226]. CuO-MnO@PEG nanocomposites disrupt copper homeostasis through GSH depletion and ATP7A/B inhibition [227]. Immunomodulatory effects are achieved via HMGB1/ATP release, enhancing DC-CTL activation when combined with PD-L1 inhibitors [228]. Chu et al. engineered DOX-loaded hollow copper ferrite nanoparticles (DOX@HCFP) with tumor microenvironment-responsive trimodal therapy (chemodynamic/photothermal/chemo), achieving 35.25% photothermal efficiency and GSH depletion-enhanced synergistic antitumor efficacy [199].

Zinc-based oxides utilize Zn^2^⁺-mediated mitophagy and β-catenin degradation for metastasis suppression. ZnO NPs inhibit OS progression through Hypoxia-Inducible Factor 1 alpha(HIF-1α)/BNIP3/LC3B pathway modulation [229]. Biodegradable Zn-0.8Li scaffolds demonstrate PI3K/Akt pathway-mediated antitumor effects alongside osteogenic activity [230]. Cheng et al. synthesized ZnONPs via green synthesis using Rehmannia extract, inducing MG-63 apoptosis through ROS generation, MMP reduction, and Bax/caspase-3/9 activation while suppressing cell adhesion to limit metastatic potential [200].

Titanium-based oxides (TiO_2_) exhibit broad-spectrum ROS generation under ultrasound/light activation. Amorphous TiO_2_/polymer hybrids enhance sonodynamic efficiency [231]. RhRu/Ti_3_C_2_T_x_ nanozymes combine photothermal conversion and catalytic H_2_O_2_ decomposition for multimodal therapy [40].

Manganese-based oxides (MONs) address tumor hypoxia while enabling theranostics. HMnO nanoparticles achieve targeted drug delivery through biomimetic coating [232]. MnO nanospikes induce ICD and cGAS-STING pathway activation for enhanced immunotherapy [233]. MONs potentiate radiotherapy by oxygen generation and ROS amplification [234]. Zhang et al. engineered GCMMR nanocomposites (GOx/Cu^2^⁺ core, MET@MnO_2_ shell, RGD-PEG coating) for synergistic starvation/chemodynamic therapy through GSH depletion, Fenton-driven OH generation, and biomineralized enzyme protection [195]. Popov et al. engineered photochromic PVP-WO_3_ nanoparticles (∼1 nm) with selective OS cytotoxicity (IC_50_:5 mg/mL) and low hMSC toxicity, while RT-PCR profiling of 96 ROS/cell-death genes revealed molecular mechanisms [204].

#### 3.4.3. Metal–Organic Framework

Metal–organic frameworks (MOFs) represent a class of porous crystalline materials formed through coordination-driven self-assembly of metal ions/clusters and organic ligands. Characterized by highly ordered networks, tunable pore sizes (0.3–20 nm), exceptional surface areas (up to 7000 m^2^/g), and abundant functional sites, their structural and functional properties can be precisely engineered through metal node and ligand selection. These attributes enable diverse applications in gas adsorption, catalysis, drug delivery, and biosensing, with particular therapeutic potential in oncology. Major MOF categories include ZIFs, MILs, UIO series, IRMOFs, and porphyrinic MOFs, differentiated by metal node composition. Du et al. engineered HA@MOF/D-Arg nanoparticles for dual-action OS radiotherapy enhancement: Fenton-mediated ROS generation and D-Arg-derived NO suppression of HIF-1α to alleviate hypoxia and boost radiosensitivity [205]. Li et al. designed Ta/Zr-co-doped TZM nanoMOFs with TCPP ligands, boosting radiotherapy via enhanced X-ray absorption and RDT efficacy through narrowed HOMO-LUMO gaps while activating antitumor immunity via ICD and enabling trimodal imaging [210].

ZIFs (Zeolitic Imidazolate Frameworks) exhibit zeolite-like topologies constructed from imidazolate ligands and divalent metal ions (Zn^2^⁺, Co^2^⁺). Their pH-responsive degradation in acidic tumor microenvironments enables controlled release of chemotherapeutics (e.g., 5-fluorouracil) and zinc ions, synergizing chemotherapy with microenvironment modulation [235,236]. Microwave-responsive ZnS@ZIF-8 composites demonstrate dual functionality in tumor ablation through thermo-chemotherapy and zinc-mediated osteogenesis [204]. SF-ZIF@NA hydrogels achieve selective osteoclast inhibition while promoting vascularized bone regeneration in osteoporotic defects [237]. Zinc ions released from ZIF-8 enhance osteogenic differentiation and angiogenesis, supporting its integration into polylactic acid bone scaffolds [238,239]. Glucose-deprivation strategies using GOx@ZIF-8/5-FU nanofibers demonstrate cascade therapeutic effects against OS [235]. Tantalum/zirconium co-doped MOFs (TZM) reverse immunosuppressive microenvironments in metastatic OS through radiosensitization and immunogenic cell death induction [210]. Ma et al. designed 3D-printed Ti scaffolds integrating ZIF-8 nanoparticles co-loading DOX and IDO inhibitor for microwave-triggered thermo-chemo-immunotherapy and Zn^2^⁺-mediated osteogenesis via immunogenic cell death activation and scaffold-guided bone regeneration [207]. Wu et al. engineered DOX-Fe_3_O_4_@ZIF-8 nanoparticles combining pH-responsive ZIF-8’s high porosity with Fe_3_O_4_’s magnetic guidability, enabling tumor-targeted DOX release under acidic conditions via external magnetic field guidance for precision chemotherapy [208]. Deng et al. engineered IrO2@ZIF-8/BSA-FA(Ce6) nanoplatform with catalase-mimicking IrO2 NPs and pH-responsive ZIF-8 for NIR/pH-triggered drug release, oxygen generation, and high-efficiency (62.1%) stable photothermal therapy [209].

MILs (Materials of Institute Lavoisier), developed using Fe/Cr clusters and carboxylate ligands, display superior thermal/chemical stability. Electronic modulation via ligand engineering enhances their nanozyme activity—NO-functionalized MIL-53(Fe) exhibits 10-fold increased oxidase activity compared to pristine counterparts, enabling ROS-mediated tumor cytotoxicity [240]. Co-doped MIL-101(Fe) improves Fenton-like catalysis for microenvironment-specific ROS generation [241]. Carbonized MIL-88C derivatives show potential as drug/gene carriers for OS targeting [242]. MIL-120(Al)-AP’s CO_2_ adsorption capacity may facilitate tumor pH modulation [243]. Wang et al. developed FA-BSA-targeted PDA-sealed Fe-MOF nanoparticles co-loaded with D-Arg/GOX/TPZ, enabling MRI-guided multimodal OS therapy through synergistic ST/CDT/GT/chemo under triple ROS/NO/free radical stress [206].

UiO frameworks, zirconium-based architectures with terephthalate linkers, demonstrate pharmaceutical stabilization capabilities. CA@UiO-66 enhances caffeic acid stability while inhibiting bacterial growth [244]. LysB/Rif@UiO-66 nanocomposites exhibit amplified antimicrobial effects through enzyme-antibiotic synergy [245]. Defect-engineered UiO-66 variants (Br-doped/aminated) improve drug loading efficiency and pollutant adsorption [246,247,248]. Current optimization focuses on hydrothermal stability enhancement for prolonged in vivo applications [249,250]. Emerging strategies combine UiO-66’s catalytic properties (H_2_/CO_2_ conversion) with antitumor functions through ROS-generating composites like UiO-66@Pt [251,252]. Chen et al. developed TPZ/PFA-loaded UiO-66@PDA nanoparticles via MOF encapsulation, utilizing high surface area/porosity for dual-drug delivery and PFA-enhanced hypoxia-triggered TPZ activation, inducing apoptosis through oxygen depletion [211].

## 4. Discussion

The rapid advancement of nanotechnology has revolutionized OS treatment by overcoming limitations of conventional therapies through multifunctional nanomaterials. This review systematically examines the functional characteristics and therapeutic applications of nanovesicles, polymers, and inorganic non-metallic/metallic materials in drug delivery, immunomodulation, catalytic therapy, thermal effects, and tissue engineering. For instance, stimulus-responsive nanocarriers enable spatiotemporal control of drug release, catalytic nanomaterials generate cytotoxic ROS via TME modulation, while biomimetic and bone-targeting strategies enhance site-specific accumulation. Immunomodulatory nanomaterials reshape immunosuppressive TME to activate antitumor immunity.

Notably, while most preclinical studies focus on OS models, emerging evidence reveals both shared efficacy and cancer-type-specific variations. Passive targeting via EPR effects, ligand-mediated active targeting, and stimulus-responsive mechanisms show broad applications in hepatocellular [253] and breast cancers [254]. Nanomaterial-mediated photothermal therapy achieves 78% tumor regression in OS xenografts, comparable to 72–80% efficacy in breast cancer PDX models [23]. However, OS-specific pathobiology fundamentally dictates unique therapeutic design requirements: Mineralized bone barriers: (1) OS requires bone-affinity strategies (e.g., hydroxyapatite-targeting ligands, polyphosphoinositol modifications) to penetrate calcified matrices, contrasting with vascular targeting in soft-tissue tumors. Hydroxyapatite-based systems exploit OS’s inherent mineral affinity, exemplified by Wu et al. HANPs showing 427% higher bone accumulation than conventional liposomes [129]. (2) Metastatic propensity management: With 85% pulmonary metastasis rates (vs. <30% in most solid tumors), nanoplatforms demand dual primary/metastatic targeting. The FePS3 nanoplatform ablates primary tumors via NIR-II photothermia while releasing anti-miR-19a to suppress lung metastasis pathways, demonstrating unparalleled “local therapy + systemic prevention” capacity [46]. (3) Immunosuppressive TME reprogramming: High Treg infiltration and PD-L1 expression necessitate nanomaterials integrating checkpoint blockade with immunogenic cell death induction. MPIRx nanoparticles co-delivering IR780/RRx-001 activate macrophage phagocytosis and M1 polarization, showing OS-specific “cold tumor” reprogramming efficacy [32]. Jiang’s IL-11Rα-targeted nanocomplex induces 3.2× greater T-cell infiltration in OS than hepatocellular models [100]. OS nanotherapy innovations thus center on bone targeting, antimetastasis, and osteoimmunomodulation synergies, contrasting melanoma’s reliance on antigen presentation [255] or leukemia’s focus on circulatory persistence [256].

Promising clinical candidates include (1) bone-targeted systems: ALN-LMWH-DOX-Lip reduces pulmonary nodules via dual targeting (ALN) and antimetastatic (LMWH) modifications, with 60% lower cardiotoxicity than free doxorubicin [68]. HA-DOPE@Lips/HNK achieves 83.8% tumor suppression with osteogenic differentiation, addressing post-resection regeneration needs [69]. (2) Stimulus-responsive platforms: pH/redox-dual responsive PTX-PLGA@[143B-RAW] achieves 90% tumor suppression via TME-triggered release and macrophage membrane camouflage [92]. (3) Immunomodulatory agents: MnO2@PA activates cGAS-STING, inducing 67.3% CD8+ T-cell infiltration in immunosuppressed models [22]. (4) Tissue-engineered systems: 3D-printed TCP-FePSe3 achieves 35.6-fold tumor reduction with concurrent bone regeneration [257]. Injectable MgO2-PEG hydrogel enables 67.6% tumor suppression via ultrasound-triggered cisplatin release and osteoinduction [113]. These platforms demonstrate mechanism specificity (targeting OS microenvironment/metastasis), validated efficacy (>70% suppression, >60% metastasis reduction), and clinical readiness (biocompatibility/degradability assessments).

Current challenges (Table 5) include scalable production of MOFs/biomimetic vesicles, long-term safety verification of metallic residues, and immunogenicity assessment of VLPs in primate models. Future directions should integrate AI-optimized nanomaterial design with TME-responsive probes, combining OS-specific targeting (bone ligands, metastasis inhibitors) with universal technologies (stimulus-responsiveness, immunomodulation) to accelerate clinical translation.

## Figures and Tables

**Table 2:** Representative paradigms of polymer nanomaterials for the treatment of OS.

Nanomaterial	Cell Line	Functional Properties	Tumor Model	Inhibition Rate	Innovation Points	Ref.
PTX-PLGA@[143B-RAW] NPs	143B	Paclitaxel delivery	Xenograft mice	60%	Hybrid cell membrane biomimetic delivery	[92]
mPEG-PaLA NP-PTX-DOX	K7M2	Drug delivery	Subcutaneous model	60%	Co-delivery, pH responsiveness	[96]
Nano-Apatinib 8P4-Apa NPs	143B SJSA1	Apatinib delivery	Carcinoma in situ	60%	solve the issue of TKI therapeutic resistance	[98]
STP-NPs/DOX	143B	DOX delivery	Carcinoma in situ	75.26 ± 4.28%	Tumor-targeting peptide, redox-responsive	[99]
GPSP nanoparticles	143B	Saporin delivery	Orthotopic model	significantly inhibited	Intracellularprotein delivery, boronated polymer	[23]
MH-PLGA- IR780 NPs	MG63 143B K7M2	PDT	Xenograft model	significantly inhibited	Homologous targeting Ferroptosis PA imaging	[97]
IL11-PDox	143B	DOX delivery	Orthotopic model	83.3%	Adjuvant chemotherapy IL-11Rα-targeting	[100]
CPCHCs/ siPLK1 NPs	MNNGHOS U2-OS	siRNA delivery	CDX tumor model	74.73%	Carbon dioxide-derived Non-viral gene vectors	[101]
MPIRx nanodrugs	K7M2 143B	SDT	Subcutaneous model	completely eradicated	TAM modulation CD47 immune checkpoint	[32]
NC-6300+aPDL1	K7M2	Combined aPD-L1	Subcutaneous model	significantly inhibited	Nano-immuno-oncology	[102]
IT-TQF NPs	143B	PTT	Metastatic models	surgical excision	imaging-guided surgery NIR-IIb fluorophore	[103]
SPN-PT	143B MG63	PTT PDT	Xenograft model	significantly inhibited	Dual-modal imaging	[104]
BINP	K7M2 143B	Biomineralization	Subcutaneous models	83.8%	Acidity-triggered bone-targeting	[105]
D@SLNP@ OSM-IR780	K7M2	Chemo- PDT	Subcutaneous model	98.9%	Mitochondria targeted	[106]
PLGA-PEG-PLGA hydrogels	Saos-2 MG-63	Localized delivery	Xenograft model	significantly inhibited	Co-delivery of DOX and CDDP	[107]
miR-29b:pBAE nanoparticles	SaOS2 K7M2	miR-29b delivery	Orthotopic model	45%	bone regenerations MicroRNAs	[108]
FSD-CHR @PPP	K7M2-Luc	siRad18 delivery	Orthotopic model	significantly inhibited	Chemotherapy resistance Cell-penetrating peptide	[20]
L-Dox/Nox4i @Gel	MNNGHOS K7M2 NIH3T3	Chemo- immunotherapy	Stroma-rich OS mouse model	significantly inhibited	Cancer-associated fibroblasts Sequential hydrogels	[109]

**Table 3:** Representative paradigms of inorganic nonmetallic nanomaterials for the treatment of OS.

Nanomaterial	Cell Line	Functional Properties	Tumor Model	Inhibition Rate	Innovation Points	Ref.
nano-HAPs	OS-732	Downregulates the signaling pathway	Xenograft model	75.4%	FAK/PI3K/Akt signaling pathway	[126]
HA–BSA–PTX NPs	143B	PTX delivery	In situ OS model	significantly inhibited	Sustained drug release Osteogenesis effects	[127]
DOX-HA complex	143B	DOX delivery	Xenograft model	significantly inhibited	Nano-hydroxyapatite nHA micro-hydroxyapatite mHA	[128]
HANPs	143B UMR106	Mitochondrial Apoptosis	Xenograft model	significantly inhibited	hydroxyapatite nanoparticles Different morphologies	[129]
HANPs	UMR106	Tumor immune microenvironment	Xenograft model	significantly inhibited	Different aspect ratios Macrophage polarization	[130]
DOX@ Se-CaP	MG63/ DXR	DOX delivery	MDR-OS model	significantly inhibited	Multidrug resistance Calcium phosphate	[131]
CaF2:Eu NPs	143B	Adjuvant radiotherapy	Orthotopic model	significantly inhibited	Migration inhibition Lung metastasis	[132]
Ce6@ZA/MSN/DOX-TK-DOXY	OS-732	PDT ROS waterfall flow	Orthotopic model	significantly inhibited	Chemotherapy sensitivity Bone targeting	[42]
MSNPCLCR/ CSCEFA	HOS	CL CR co-delivery	——	——	Active cancer targeting Natural agent	[133]
SeNP-MSNs	SaoS-2	Se delivery	——	——	induces ROS cell apoptosis	[134]
MCD nanoparticles	143B U2OS	PTT Cuproptosis	Orthotopic model	69.7%	Mild photo-thermal therapy Neoplastic bone destruction	[135]
DOX-Se/MBGnanospheres	MG63	DOX-Se delivery	——	——	Filling biomaterial Bone tissue engineering	[136]
Cs-g-PCL/MBGs/Cisplatin	MG-63	Cisplatin delivery	——	——	Magnetic bioactive glasses Nanofiber	[137]
Mesoporous Carbon Nps	HOS	Drug delivery	——	——	Self-immolative coating Controlled release	[138]
PEG-GO-FA/ICG NPs	U2 MG63 SaOS-2	MTH1 inhibitor	Xenograft model	significantly inhibited	ER stress Chemo-PDT	[139]
GO@PEG-Pt	MG63 SAOS-2	Cisplatin-based drug delivery	——	——	Platinum-based drug Nanomedicine	[140]
BPQDs-DOX @OPM	Saos-2	PTT DOX delivery	Xenograft model	significantly inhibited	Hybrid membrane	[141]
Carbon- BNQDs	MG-63	Photothermal	——	——	One-pot synthesis Anti-inflammatory Antioxidant	[142]
F127-BG -BPQDs	Saos-2	Tissue regeneration Microwave treatment	——	——	Bioactive glass Theragenerative	[143]

“——” means that no animal experiments were conducted for this reference.

**Table 4:** Representative paradigms of metal-based nanomaterials for the treatment of OS.

Nanomaterial	Cell Line	Functional Properties	Tumor Model	Inhibition Rate	Innovation Points	Ref.
anti-miR-19a/FePS@PPF	HOS MG63	PTT miRNA delivery	Xenograft model	completely eradicated	FePS3 nanoplatform Gene therapy	[46]
Au NPs	MG63	Apoptosis	——	50%	Size-dependent cytotoxicity	[192]
AuNR@CA nanohybrids	Saos-2	Steerable hyperthermia	Xenograft model	73.5%	Chlorogenic acid Bone regeneration	[193]
F-AgÅPs	143B SJSA-1	Inhibiting PDK	Subcutaneous models	49.19%	Glucose metabolism ROS-dependent apoptosis	[196]
Silver nanoparticles	MG-63	Cytotoxic	——	significantly inhibited	Rhizophora apiculata Tannin	[197]
AgBiS_2_ NPs	UMR-106	CT imaging phototherapy	Subcutaneous model	completely inhibited	synergistic photodynamic bioimaging properties	[198]
DOX@ HCFP NPs	143b	DOX delivery CDT PTT	Xenograft model	completely inhibited	hollow copper ferrite nanospheres	[199]
FeS_2_@ CP NPs	U2 HOS	PTT CDT	Xenograft model	92.44%	Pyrite nanoparticle Fenton catalyst	[39]
W-TiO_2_ nanorods	143B	SDT CDT TME-regulating	Xenograft model	completely eradicated	GSH depletion Tumor microenvironment	[43]
ZnONPs	MG-63	ROS apoptosis	——	significantly inhibited	Rehmanniae radix green synthesized	[200]
AS-ZnONPs	MG-63	Apoptosis	——	significantly inhibited	Green synthesized	[201]
FSR-Fin56	MNNG/ HOS	Ferroptosis PTT CDT	Subcutaneous models	significantly inhibited	iron-based nanovehicle LPOs	[202]
DOX/Fe_3_O_4_@PMMA	143B	MH ablation DOX delivery	Xenograft model	91.8%	PMMA bone cement Magnetic hyperthermia	[203]
WO3 NPs	MNNGHOS	High cytotoxicity	——	50%	Ph-sensitive PVP-stabilized	[204]
MnO2@ PA NPs	U2OS 143B	MRI Bone targeting	Xenograft model	significantly inhibited	Phytic acid targeting therapy	[22]
mCu&Ce@ ICG/RGD	143B	PTT-CDT-ICD	Subcutaneous model	significantly inhibited	NIR II fluorescent/ MR bio-imaging	[171]
HA@MOF/ D-Arg	K7M2	Radiotherapy	Subcutaneous model	significantly inhibited	Metal–organic frameworks Hypoxia	[205]
DArg/GOX/TPZ@MOF(Fe)PDA/Fe^3+^/FA-BSA	143B	RT ST GT CDT MRI Chemotherapy	Subcutaneous model	significantly inhibited	Starvation therapy Gas therapy	[206]
Ti-ZIF-8@ DOX-IDO (TZDI)	K7M2	Immunotherapy Microwave ablation	Orthotopic model	significantly inhibited	Microwave thermo-chemotherapy	[207]
DOX-Fe_3_O_4_ @ZIF-8(DFZ)	K7M2	DOX delivery	Subcutaneous model	83.22%	PH-responsive magnetic nanoparticles	[208]
IrO2@ZIF-8/BSAFA Ce6	MNNGHOS	PTT PDT	Subcutaneous model	significantly inhibited	Iridium oxide dual-stimulus-responsive	[209]
Porous TZM	K7M2	RT–RDT immunotherapy	Metastasis model	completely eradicated	X-ray radiosensitizer anti-PD-L1 treatments	[210]
TPZ/PFA@UiO-66@PDA	143B	PTT TPZ/PFA delivery	Subcutaneous model	71%	Hypoxia-activated Metal–organic framework	[211]
TOhAu@Pt- PEG-Ce6/HA	LM-8	PTT PDT oxygen production	Subcutaneous model	significantly inhibited	Anisotropic plasmon resonance	[194]
GOxCuCaP@MNSsMET@PEG-RGD	MNNG/ HOS	starvation therapy CDT	Subcutaneous model	85.64%	Biomineralization Glutathione depletion	[195]

“——” means that no animal experiments were conducted for this reference.

**Table 5:** Comparison of advantages and limitations among different types of nanomaterials.

Representative Nanomaterials	Advantages	Limitations
Liposome [258]	Structural flexibility	Poor stability
	Biosafety	Short circulating half-life
	Biphasic drug-carrying capacity	Limited drug encapsulation
Exosome [259]	Crossing the biological barrier	Difficult to purify and low output
	Natural targeting tendency	Biological function unknown
	Endogenous signaling properties	Significant heterogeneity
Poly(lactic-co-glycolicacid) (PLGA) [260]	Degradability	Uneven particle size distribution
	Scale-up potential	Solvent residue problems
	High in vitro stability	Limitations of hydrophobic matrices
	Controlled circulation in vivo	Differences in cellular uptake efficiency
Hydrogel [261]	High water absorption and retention	Insufficient mechanical properties
	Tunable mechanical properties	Functionalization Limitations
	Injectability and moldability	Poor degradation controllability
Hydroxyapatite (HA) [262]	Osteoconductivity and osteoinductivity	Brittleness and low toughness
	Bionic properties and highly processable	Poor fatigue resistance and pyrolysis risk
	Morphological diversity	High machining difficulty
Mesoporous silica (MSN) [263]	Tunable pore size and pore structure	Non-specific adsorption problems
	Inert silica matrix	Brittleness and susceptibility to collapse
	Proven preparation process	Problems with clogged orifices
	Cost-controllable	Activity of surface silica hydroxyl groups
Graphene oxide (GO) [264]	Highly chemically modified simple process	Poor dispersion and mechanical anisotropy
	Good thermal and electrical conductivity	Limited water solubility
	Easily available raw materials, low cost	Limited thermal stability
Gold nanoparticles (Au NPs) [265]	Surface plasmon resonance effect	Aggregation and stability issues
	Scattering catalytic activity	Restricted excretory pathways
	High efficiency of photothermal conversion	High cost of precious metals
Metal–organic frameworks (MOFs) [266]	Recyclability and recycling potential	Production costs and process complexity
	Optical and magnetic properties	Mass transfer resistance and functional
	Multi-field coupling function	metal ion toxicity and low impurity tolerance

## Data Availability

No new data were created or analyzed in this study. Data sharing is not applicable to this article.

## Abbreviations

The following abbreviations are used in this manuscript:

OS	Osteosarcoma
EPR	Enhanced Permeability and Retention
mTOR	Mammalian Target of Rapamycin
CAR-T	Chimeric Antigen Receptor T cell
PD-1	Programmed Cell Death Protein 1
DOX	Doxorubicin
miRNA	MicroRNA
HIF-1α	Hypoxia-Inducible Factor 1 alpha
pH	Power of Hydrogen
ROS	Reactive Oxygen Species
CDT	Chemodynamic Therapy
PDT	Photodynamic Therapy
PTT	Photothermal Therapy
MOFs	Metal–Organic Frameworks
PLGA	Poly(lactic-co-glycolic acid)
PDA	Polydopamine
TCP	Tricalcium Phosphate
MRI	Magnetic Resonance Imaging
CT	Computed Tomography
CD47	Cluster of Differentiation 47
PD-L1	Programmed Death-Ligand 1
siRNA	Small Interfering RNA
